# Mitophagy-related gene TRIP13 predicts prognosis and immune response and promotes proliferation and migration *in vitro* and *in vivo* of clear cell renal cell carcinoma

**DOI:** 10.3389/fphar.2025.1736086

**Published:** 2025-12-18

**Authors:** Zhongjun Jiang, Lanlan Wang, Zhongrun He, Lian Guo, Wen Luo, Ying Fu, Qiyu Xiao, Guanglan Chen, Yinzi Liu

**Affiliations:** 1 Department of Nuclear Medicine, The Affiliated Cancer Hospital of Xiangya School of Medicine, Central South University / Hunan Cancer Hospital, Changsha, China; 2 Department of Thyroid and Breast Surgery, The Affiliated Nanhua Hospital, Hengyang Medical School, University of South China, Hengyang, China; 3 Department of Diagnostic Radiology, The Affiliated Cancer Hospital of Xiangya School of Medicine, Central South University / Hunan Cancer Hospital, Changsha, China; 4 Department of Pharmacy, Zunyi Medical University, Zunyi, China; 5 Department of Cardiopulmonary Function Test Center, The Affiliated Cancer Hospital of Xiangya School of Medicine, Central South University / Hunan Cancer Hospital, Changsha, China; 6 Department of Clinical Laboratory Center, The Affiliated Cancer Hospital of Xiangya School of Medicine, Central South University / Hunan Cancer Hospital, Changsha, China

**Keywords:** clear cell renal cell carcinoma, immune response, mitophagy, prognosis, TRIP13

## Abstract

**Background:**

The incidence of clear cell renal cell carcinoma (ccRCC) is increasing every year. Mitophagy is a unique form of autophagy that plays a crucial role in cancer development and invasion. However, its role in ccRCC remains to be fully elucidated.

**Methods:**

After extracting mitophagy-related genes (MRGs), differential expression analysis was performed to screen differentially expressed genes (DEGs). Univariate Cox regression analysis was used to screen prognostic-related DEGs, CNV mutation frequencies were compared, and consensus cluster analysis was constructed to evaluate the survival and functional enrichment status among different subtypes. LASSO Cox regression analysis was used to identify key prognostic genes and construct risk models to evaluate the prognostic value and immune contribution. The protein and mRNA expression levels of independent prognostic genes and their effects on ccRCC function were verified by *in vitro* and *in vivo* experiments.

**Results:**

The study found 174 DEGs, including 9 prognosis-related DEGs. These 9 DEGs were used to cluster ccRCC patients into two subtypes. Significant differences existed between the two subtypes in the survival status and KEGG functions. Finally, three core genes (JUP, TRIP13, and ACAD11) were identified for constructing a risk model, which can accurately predict the prognosis of ccRCC patients and evaluate the immune status. TRIP13 was identified as a key independent prognostic gene for ccRCC, and its protein and mRNA expression levels were highly expressed in ccRCC. ccRCC growth and motility can be markedly inhibited by TRIP13 knockdown, which also increases their susceptibility to destruction by CD8^+^ T cells.

**Conclusion:**

The prognosis and immune response of patients with ccRCC could be reliably estimated by the model in our cohorts created using MRGs in this research. The development of ccRCC is significantly influenced by MRGs, particularly TRIP13. This study can assist in offering ccRCC patients individualized treatment options.

## 1 Introduction

Kidney cancer ranks among the top six genitourinary malignancies in males worldwide, accounting for approximately 5% of all male cancers (Sieg et al., 2022). According to statistics, the number of kidney cancer cases has increased year by year and has reached 74,000 cases by 2020 (Sung et al., 2021). Renal cell carcinomas constitute more than 90% of kidney cancer cases, with clear cell renal cell carcinoma (ccRCC) being the most prevalent subtype (Rose and Kim, 2024; Chen Z. X. et al., 2025). Epidemiology shows that thousands of people die from ccRCC every year. The etiology of ccRCC is multifaceted, and the high degree of tumor tissue heterogeneity poses significant challenges for both diagnosis and treatment. The pathophysiology of ccRCC remains incompletely understood, and effective tumor biomarkers have yet to be identified (Yin et al., 2019). Therefore, identifying additional prognostic markers is crucial for improving the clinical management and prognosis of patients with ccRCC.

Mitophagy, a specialized form of autophagy, is a critical cellular process that eliminates damaged or excess mitochondria through autophagic lysosomes, thereby maintaining mitochondrial quality and cellular homeostasis (Zhao et al., 2024; Wang et al., 2024; Bunu et al., 2025; Lin et al., 2024). This process is frequently triggered by oxidative stress or elevated bioenergetic demand, both of which are crucial for cancer initiation and invasion (Bernardini et al., 2017; Fan et al., 2025; Zhi and Wang, 2024). Interestingly, various mitophagy pathways seem to have tumor-promoting and tumor-suppressing effects in cancer (Poole and Macleod, 2021). In hypoxia and metabolic stress, mitochondrial recycling and reduction of oxygen consumption are achieved by activating mitophagy, which increases tumor cell survival (Vara-Perez et al., 2019). Our previous study established the mitophagy-related gene CHDH as an independent prognostic factor in oral squamous cell carcinoma (OSCC) and demonstrated that its overexpression suppresses tumor progression and immune evasion, highlighting its potential as a novel therapeutic target (Chen L. et al., 2025). In recent years, related articles have also reported the role of mitophagy in ccRCC, exploring the impact of MRGs on tumor biological behavior or clinical prognosis. Lai Jiang and his team revealed the spatial distribution characteristics of MRGs in ccRCC tissues and their effects on cell communication through single-cell analysis combined with spatial transcriptomics (Jiang et al., 2024). Hang Yin constructed an MRGs prognostic model by integrating genomics and single-cell analysis, and clarified the predictive performance of the prognostic model (Yin et al., 2024). Although some studies have reported the role of MRGs in ccRCC, its research is still far from enough.

Thyroid hormone receptor interacting protein 13 (TRIP13) is an AAA + ATPase involved in cell cycle regulation and genome stability. It is highly expressed in a variety of tumors (such as ccRCC) and is associated with tumor progression and poor prognosis (Bunu et al., 2025). TRIP13 is a gene related to mitochondrial autophagy. Studies have found that TRIP13, as MRGs, constructs the prognostic characteristics of multiple myeloma and can be used as a prognostic marker for multiple myeloma (Lv and Zhang, 2024). In ccRCC, high expression of TRIP13 is an independent poor prognostic indicator for ccRCC survival, which may be an important driving factor for systemic treatment resistance in renal cancer, but its specific mechanism is still unclear (Chen X. et al., 2025). Therefore, studying how TRIP13 regulates the development of ccRCC through mitochondrial autophagy will not only help to reveal the pathogenesis of ccRCC, but may also provide new ideas for the development of therapeutic strategies targeting TRIP13 or combining mitochondrial autophagy inhibitors.

Therefore, this study investigated the immunological and prognostic associations of MRGs in ccRCC using data from the TCGA and GEO datasets. TRIP13 was further validated through *in vitro* and *in vivo* experiments, shedding light on the role of MRGs in ccRCC. The findings of this research offer potential new treatment strategies for clinicians and provide fresh insights and directions for future studies in this field.

## 2 Materials and methods

### 2.1 Raw data download and preprocessing

Transcriptome profiles and corresponding clinical information for 537 patients with ccRCC were downloaded from The Cancer Genome Atlas (TCGA) database (accessed 26 April 2024). Additionally, the GSE29609 dataset, containing 39 ccRCC tumor samples with clinical annotations, was obtained from the Gene Expression Omnibus (GEO) database. TCGA RNA-seq data were converted to fragments per kilobase per million reads (FPKM) and log2-transformed, whereas GEO data underwent quantile normalization followed by log2 transformation. After quality control, the two datasets were merged for downstream analyses using the “limma” R package. Mitophagy-related genes (MRGs) were retrieved from the GeneCards database (Rouillard et al., 2016) using “Mitophagy” as the keyword. Genes with a correlation score >1 were selected as the genes we studied. A detailed list of genes is provided in Supplementary Table S2.

### 2.2 Identification of differentially expressed genes and CNV analysis

Differentially expressed genes (DEGs) between tumor and normal tissues were identified using the “limma” package with thresholds of |log2 fold change| ≥ 1 and *FDR* < 0.05. Prognosis-related DEGs were then screened by univariate Cox regression analysis using the “survival” and “survminer” packages. Gene-gene correlation networks were visualized using “igraph” and “RColorBrewer”. Copy number variation (CNV) profiles for ccRCC were obtained from the UCSC Xena platform, and CNV alterations of prognostic MRGs were visualized using the “RCircos” package.

### 2.3 Consensus clustering of MRG expression patterns

To identify molecular subtypes based on MRG expression, consensus clustering was performed using the “ConsensusClusterPlus” package with resampling (1,000 iterations). Principal component analysis (PCA) was applied to evaluate subtype separation. Kaplan-Meier survival curves were generated to compare survival outcomes between subtypes. Heatmaps and boxplots were created with “pheatmap” and “ggplot2”, respectively, to visualize gene expression profiles and clinicopathological features.

### 2.4 Pathway enrichment analysis of MRGs

GSVA (Gene Set Variation Analysis) is an advanced computational method that leverages gene set enrichment techniques to perform in-depth analysis and interpretation of gene expression data (Hänzelmann et al., 2013). Through GSVA, we can identify pathway-level differences between subtypes, revealing distinct biological activities and functional variations. Additionally, Gene Set Enrichment Analysis (GSEA) provides a powerful tool to investigate specific chromosomal regions, enriched pathways, and genomic activities, offering deeper insights into the molecular mechanisms underlying subtype-specific behaviors. Together, these methods enable a comprehensive understanding of the functional and genomic landscape associated with different subtypes (Cao et al., 2019; Subramanian et al., 2005). Using GSEA, we identified pathways that are either active or suppressed in subtype B. To further explore the unique biological pathways associated with these MRG groups, we performed the KEGG (Kyoto Encyclopedia of Genes and Genomes) pathway analysis. This was accomplished using the R packages “limma”, “GSEABase”, “GSVA”, and “clusterProfiler”. Unless otherwise specified, enrichment results were evaluated using FDR-adjusted *q*-values, with *q* < 0.05 regarded as significant. This approach allowed us to uncover subtype-specific pathway activities and gain deeper insights into the functional mechanisms underlying these MRG groups.

### 2.5 Construction of the mitophagy-related prognostic model

First, we identified the prognostic MRGs using univariate Cox regression analysis. Then ccRCC patients were randomly divided into training and testing groups. The prognostic model was constructed using samples from the training group, and its accuracy was verified using samples from the testing group. To refine the selection of prognostic factors, we employed LASSO (least absolute shrinkage and selection operator) regression using the R package “glmnet”, which is a complex statistical method for analyzing high-dimensional data and prevents overfitting. The best multivariate Cox model was determined by cross-validating the feature MRGs and applying the optimization criterion of minimum error. Based on the median risk score, both the training and test groups were categorized into high-risk and low-risk subgroups, enabling the evaluation of the model’s predictive performance. We created pertinent ROC curves and calculated the area beneath the curve (AUC) using the 3 R packages “survival”, “survminer” and “timeROC” to assess the risk model. Before determining the clinical significance of independent prognostic factors, we assessed the relationship between clinical characteristics and patient outcomes using multivariate Cox regression analysis. By combining the risk score with other clinicopathological indicators, the overall survival of patients can be accurately assessed. The clinicopathological characteristics and risk scores of patients were scored, and a total score was summarized to reflect the 1–5 years survival rate of patients. Calibration curves were used to evaluate the agreement between predicted and observed survival probabilities, while decision curve analysis (DCA) assessed the clinical utility of the nomogram.

### 2.6 Immune cell infiltration analysis

The CIBERSORT algorithm uses the principle of linear support vector regression to deconvolve the immune cell subtype expression matrix to estimate the abundance of immune cells (Newman et al., 2015). To examine the prevalence of various immune cell types in the low-risk and high-risk groups, CIBERSORT was applied to estimate the infiltration level and proportion of 22 immune cells with 1,000 permutations. The differences between the low-risk and high-risk groups were visualized using the “ggplot2” package in R. Additionally, the ESTIMATE (Estimation of STromal and Immune cells in MAlignant Tumours using Expression data) algorithm was utilized to quantify immune cell infiltration (ImmuneScores) and stromal cell presence (StromalScores), as well as to assess the overall tumor microenvironment. This comprehensive approach provided insights into the immune landscape and stromal composition within the tumor microenvironment across different risk categories.

### 2.7 Drug sensitivity prediction

The potential sensitivity of ccRCC samples to anticancer agents was evaluated using “oncoPredict”, based on half-maximal inhibitory concentration (IC50) values derived from the Genomics of Drug Sensitivity in Cancer (GDSC) database. Additionally, we employed the R package “ggplot2” to create detailed and visually compelling representations of all statistical analyses, enhancing the interpretability and presentation of our findings. This approach allowed us to provide actionable insights into potential therapeutic strategies for ccRCC patients based on their risk profiles.

### 2.8 Screening and validation of independent prognostic genes

Through univariate and multivariate Cox regression analysis, forest plots were generated using the R package “forestplot” to visualize each variable’s *p*-value, hazard ratio (HR), and 95% confidence interval (CI). This allowed us to identify genes capable of independently predicting prognosis. The R package “ggplot2” was employed to compare the expression levels of independent prognostic genes between ccRCC tissues and adjacent normal tissues. Kaplan-Meier survival curves were constructed to compare high and low expression levels of these genes, with statistical significance assessed using the log-rank test and univariate Cox regression to derive *p*-values and HR with 95% CI. A *p*-value of less than 0.05 was considered statistically significant, ensuring robust identification of prognostic biomarkers. To investigate biological network integration for gene prioritization and prediction of independent prognostic gene functions, the GeneMANIA prediction server (http://www.genemania.org, accessed on 15 March 2025) was used in this study.

### 2.9 Human tissue samples

Twelve pairs of ccRCC and adjacent non-tumor tissues were collected from the Affiliated Cancer Hospital of Xiangya School of Medicine, Central South University (January–August 2024). All samples were immediately preserved and processed for molecular and histological analyses. Informed consent was obtained from all participants.

### 2.10 Immunohistochemistry

After fixing the ccRCC and adjacent tissues with 4% paraformaldehyde, they were embedded in paraffin and sectioned to a thickness of 6 μm. The sections were then dewaxed and rehydrated for immunohistochemical staining. Antigen retrieval was performed using Tris-EDTA buffer (10 mM Tris HCl, 1 mM EDTA), and endogenous peroxidase activity was quenched. The sections were boiled in a pressure cooker for 5 min, followed by three washes. BSA (bovine serum albumin) was applied to block nonspecific binding, and the sections were incubated with TRIP13 primary antibody overnight at 4 °C. On the following day, the sections were incubated with secondary antibodies for 1 h at room temperature. Finally, the nuclei were counterstained with hematoxylin, and the sections were dehydrated and mounted with neutral resin for microscopic examination.

### 2.11 Quantitative real-time PCR

Based on experimental requirements, we synthesized complementary DNA (cDNA) using a cDNA reverse transcription kit manufactured by Shanghai Regeneron and extracted total RNA using the RNeasy Mini Kit from QIAGEN (Beijing). PCR reactions were performed according to the manufacturer’s recommended protocol using TaqMan Gene Expression Master Mix provided by Shanghai Bio-Rad, along with human TRIP13 and GAPDH-specific TaqMan probes designed by Shanghai Sangon Biotech. These reagents and protocols ensured the accuracy and reliability of the results.

### 2.12 Western blot

Forty-eight hours after cells were transfected with TRIP13 or a control plasmid, total protein was extracted using RIPA lysis buffer (ThermoFisher, 89,900) for subsequent analysis. Protein lysates were separated by SDS-PAGE electrophoresis and transferred to a PVDF membrane. The membrane was washed with TBST (50 mM TRIS, 150 mM sodium chloride, 0.1% Tween 20, pH 7.4) and blocked with 5% skim milk in TBST for at least 1 h at room temperature. Subsequently, the target protein was incubated with a primary antibody specific to TRIP13 overnight at 4 °C, followed by incubation with an HRP-conjugated secondary antibody (goat anti-rabbit) for 1 h at room temperature. Visualization was performed using the ChemiDoc imaging system. Band intensity was quantified using ImageJ software to assess TRIP13 expression levels.

### 2.13 MTT

Before transfection, 2 × 10^5^ cells were seeded into six-well plates and cultured at 37 °C for 24 h until the cells reached 30%–40% confluency. Subsequently, 1 mL of culture medium containing TRIP13 knockdown lentivirus and 40 μL of transfection reagent were added to each well. After 12 h, the culture medium was replaced with normal culture medium and cultured for a further 24 h 72 h after transfection, untransfected cells were eliminated with puromycin. The selected cells were seeded into 96-well plates at a density of 6,000 cells per well and cultured for 72 h 50 μL of MTT solution (2 mg/mL) was added to each well and incubated for another 4 h. After aspirating the supernatant, 150 μL of DMSO was added and dissolved with shaking for 10 min. The absorbance of each well was measured at 490 nm using a microplate reader.

### 2.14 Colony formation assay

Before transfection, 2 × 10^5^ cells were seeded in six-well plates and cultured for 24 h to achieve a confluency of 30%–40%. Subsequently, 1 mL of culture medium containing lentivirus and 40 μL of transfection reagent was added to each well to knock down TRIP13 expression. Twelve hours after transfection, the culture medium was replaced with normal culture medium and cultured for an additional 72 h. Unsuccessfully transfected cells were then removed with puromycin. Surviving cells were replated at 1,000 cells per well in 24-well plates. After 6–8 days of culture, cells were fixed with 10% formaldehyde for 20 min, washed twice with PBS, stained with 0.1% crystal violet for 20 min, washed twice with PBS, air-dried, and photographed to calculate colony formation efficiency.

### 2.15 Scratch assay

Before transfection, 2 × 10^5^ cells were seeded in a six-well plate. When cells reached 30%–40% confluency, 1 mL of Opti-MEM medium containing lentivirus and 40 μL of transfection reagent was added to each well to knock down TRIP13 expression. Twelve hours after transfection, the culture medium was replaced with normal medium and cultured for an additional 72 h. Unsuccessfully transfected cells were then removed with puromycin. Unsuccessfully transfected cells were then removed using puromycin. Surviving cells were repeated at a density of approximately 3 × 10^5^ cells per well in 12-well plates and cultured until a monolayer formed. A 200 μL pipette tip was then used to create a scratch on the monolayer, and detached cells were gently rinsed with PBS. The culture medium was then replaced with serum-free medium and cultured for further analysis. Microscopic images were taken at 0 and 24 h after the scratch to analyze cell migration.

### 2.16 Transwell assay

Before transfection, 2 × 10^5^ cells were seeded in a six-well plate. When cells reached 30%–40% confluency, 1 mL of Opti-MEM medium containing lentivirus and 40 μL of transfection reagent was added to each well to knock down TRIP13 expression. Twelve hours after transfection, the culture medium was replaced with normal medium and cultured for an additional 72 h. Unsuccessfully transfected cells were then removed with puromycin. Surviving cells were harvested, resuspended in serum-free medium, and counted. 200 μL of serum-free medium containing 40,000 cells was added to the upper chamber of the Transwell; 600 μL of medium containing 10% serum was added to the lower chamber to establish a chemotactic gradient. The volume and composition of the medium were strictly controlled in each well to ensure experimental consistency. After 24 h of cell migration, cells on the membrane surface of the lower chamber were fixed with formaldehyde to preserve morphology and then stained with crystal violet. After removing unmigrated cells from the upper chamber, the cells were observed under a microscope and photographed. ImageJ was used to count the cells and calculate the cell migration rate.

### 2.17 *In vivo* tumor models

Study design and animals: Female nude mice (BALB/c strain, 5–6 weeks old) were purchased from Skajingda Biotechnology Company and housed in a specific pathogen-free environment under controlled conditions (temperature: 22 °C–26 °C, humidity: 55%, 12/12-h light/dark cycle). Mice were housed five per cage with *ad libitum* access to food and water. The experimental unit was the individual animal. Sample size and randomization: A total of 10 mice were randomly allocated into two experimental groups (shCtrl and shTRIP13-2, n = 5 per group). The sample size was determined based on preliminary data and literature reports of similar *in vivo* studies, providing adequate power to detect significant differences in tumor growth. Group allocation was performed using a computer-generated random number sequence. The investigator responsible for the allocation was different from those performing the subsequent measurements and analyses. Procedures and blinding: To establish the xenograft model, 2 × 10^6^ A498-shCtrl or A498-shTRIP13-2 cells suspended in phosphate-buffered saline were subcutaneously injected into the right flank of each mouse. The investigators measuring tumor size and body weight were blinded to the group allocation throughout the experiment. Tumor size was measured daily with a caliper once palpable, and volume was calculated using the formula: V = 1/2 × (long diameter) × (short diameter) (Sung et al., 2021). The humane endpoint for tumor burden was set at 1,000 mm^3^. Body weight, overall health, and behavior were monitored regularly. Any signs of distress were documented and addressed accordingly. Euthanasia and statistical analysis: At the end of the experiment, mice were euthanized by inhalation of 5% isoflurane until loss of consciousness, followed by carbon dioxide asphyxiation to ensure cessation of vital signs. All data were analyzed using GraphPad Prism software (version 9.0). Tumor weight and immunohistochemistry quantification data were compared between the two groups using an unpaired, two-tailed Student’s t-test. Data are presented as mean ± standard deviation (SD). A *p*-value of less than 0.05 was considered statistically significant.

### 2.18 CD8^+^ T cell cytotoxic assay

Obtained through the method outlined in the reference, the CD8^+^ T cells represent a crucial component of our research (Shen et al., 2022). Start by transfecting tumor cells with either the TRIP13 knockdown plasmid or the control plasmid, packaged in lentivirus, and let them incubate for 24 h. Following this incubation time, plate the cells at a density of 10,000 cells per well on a 96-well plate. The 96-well plate was then filled with CD8^+^ T cells following a 12-h incubation period. After co-incubating the tumor cells for 48 h, remove the culture medium and give the cells two PBS washes to remove the T cells. Lastly, the MTT test will evaluate how well CD8^+^ T cells eradicate tumor cells.

### 2.19 Knockdown of TRIP13 by transfection of lentiviral vectors

The TRIP13 knockdown was achieved using lentiviral vectors designed by GeneChem Co. Ltd. (Shanghai, China). Specifically, shCtrl, shTRIP13-1, and shTRIP13-2 were used for knockdown. TRIP13 targeting oligonucleotides are shown in Table 1. Cells were seeded in 6-well plates at a density of 2 × 10^5^ per well. After 24 h, lentiviruses were introduced along with 1 mL of medium without serum and 40 μL of transfection reagent for another 12-h incubation. After washing with PBS, the medium was replaced with 2 mL of complete medium for 72 h. Finally, puromycin was added to eliminate untransfected cells. Both WB and PCR results showed that the knockout efficiency was more than 75%.

**TABLE 1:** The targeting oligos of TRIP13.

ShTRIP13	Sequences
ShTRIP13-1	GUA​CCG​AUA​UGG​CCA​AUU​A
ShTRIP13-2	GCA​AAU​CAC​UGG​GUU​CUA​C

### 2.20 Statistical analysis

All statistical analyses were conducted in R (versions 4.3.3 and 4.4.0). Comparisons between two groups were performed using the Student’s t-test or one-way ANOVA as appropriate. Survival analyses used Cox regression and Kaplan-Meier methods with log-rank tests. *p* < 0.05 was considered statistically significant.

## 3 Results

### 3.1 Research process

To locate MRGs in ccRCC, we collected 537 ccRCC samples from the TCGA-KIRC database and added 39 tumor samples from the GEO-GSE29609 database. We identified a total of 1,686 MRGs (correlation score >1) from the GeneCards platform. Through differential expression analysis, we further pinpointed 174 MRGs that were significantly differentially expressed, providing a focused set of genes for subsequent investigation into their roles in ccRCC and other related conditions. Univariate Cox regression analysis showed that from 174 differentially expressed MRGs, 9 genes were associated with ccRCC prognosis. Our functional enrichment analysis and ccRCC consensus clustering were centered around these nine genes. Using proportional hazards model analysis and LASSO regression, three genes were chosen for the prognostic model and immune-related function analysis. TRIP13 was found to be an independent predictive gene for ccRCC by univariate and multivariate regression analysis. To confirm our results, we thoroughly validated TRIP13’s functionality *in vitro*; the whole process is depicted in Supplementary Figure S1.

### 3.2 Acquisition and CNV analysis of differential MRGs in ccRCC

**FIGURE 1:**
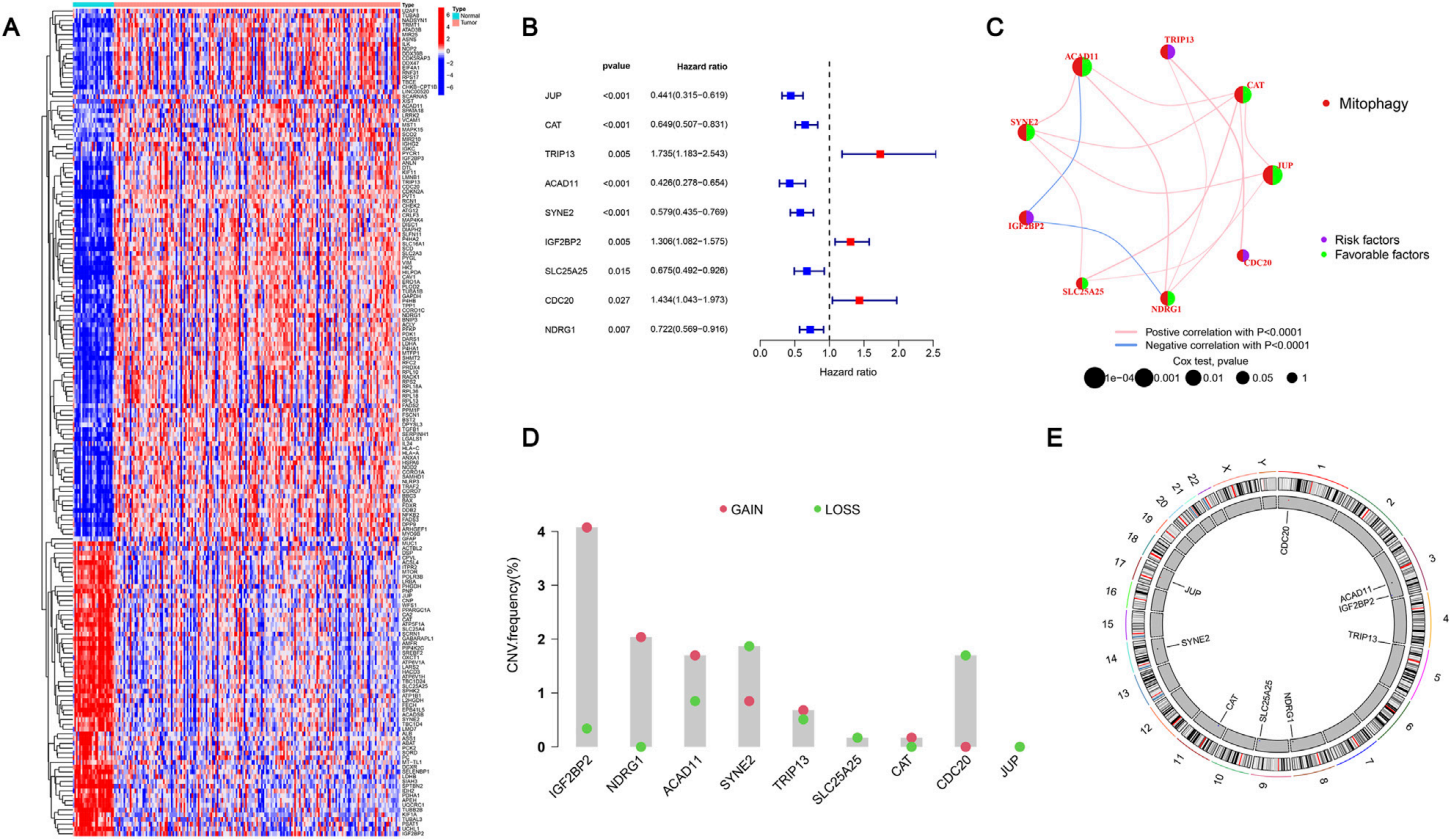
Acquisition and CNV analysis of differential MRGs in ccRCC. **(A)** Heatmap displaying the 174 differentially expressed mitophagy-related genes (DEGs) between ccRCC and normal tissues, identified using |logFC| ≥ 1 and *FDR* < 0.05. Red indicates upregulated genes, and blue indicates downregulated genes. **(B)** Forest plot summarizing the hazard ratios (HRs), 95% confidence intervals (CIs), and p-values of the nine prognostic MRGs identified by univariate Cox regression analysis. **(C)** Network diagram illustrating the correlations and potential functional associations among the nine prognostic MRGs. **(D)** Copy number variation (CNV) frequencies of the nine MRGs in ccRCC samples. Red represents copy number gains, and green represents copy number losses; bar height reflects the magnitude of the alteration. **(E)** Genomic distribution of the nine MRGs across the 23 human chromosome pairs.

First, by performing MRGs differential expression analysis on ccRCC samples and normal tissue samples in the TCGA-KIRC dataset, a total of 174 differentially expressed MRGs were identified. We call these genes differentially expressed genes (DEGs), and these DEGs were displayed by heat maps, of which 112 genes were highly expressed in ccRCC and the remaining 62 genes were highly expressed in normal tissues (Figure 1A). To further illustrate the differential expression results, a volcano plot was generated (Supplementary Figure S2A). To further explore the role of these DEGs in tumors, we used these 174 DEGs for GO and KEGG enrichment analysis. In GO, these genes are mainly involved in response to oxygen levels, ribose phosphate metabolic process, cell-substrate junction and other processes (Supplementary Figure S2B). In KEGG, they are mainly involved in the HIF-1 signaling pathway, the Glucagon signaling pathway, PPAR signaling pathway and other processes (Supplementary Figure S2C), all of which are related to the occurrence and development of tumors. After that, to obtain more accurate MRGs characteristics, we screened 9 prognosis-related DEGs (JUP, CAT, TRIP13, ACAD11, SYNE2, IGF2BP2, SLC25A25, CDC20, NDRG1) related to the prognosis of ccRCC by univariate Cox regression analysis. Figures 1B,C display the findings. Among these 9 MRGs, six genes, including JUP and CAT, are marked in blue, with HR < 1 being a favorable prognostic factor. A thorough summary of the relationships, correlations, and predictive power of MRGs is given in Figure 1C. In order to further study the mutation of the 9 genes, we used the ccRCC copy number mutation data for analysis and found that the frequency of copy number increase of IGF2BP2, NDRG1, ACAD11, CAT and TRIP13 was greater than the frequency of copy number loss, and the overall performance was copy number amplification, while the remaining four genes showed copy number loss (Figure 1D). In addition, the mutation positions of these 9 genes on 23 pairs of chromosomes were displayed by circle diagrams (Figure 1E). This visualization method helps to accurately locate key genes and pathways related to ccRCC research and provides an important basis for in-depth exploration of genetic factors that affect the occurrence of the disease.

### 3.3 Analyzing immunological responses and consensus clustering

**FIGURE 2:**
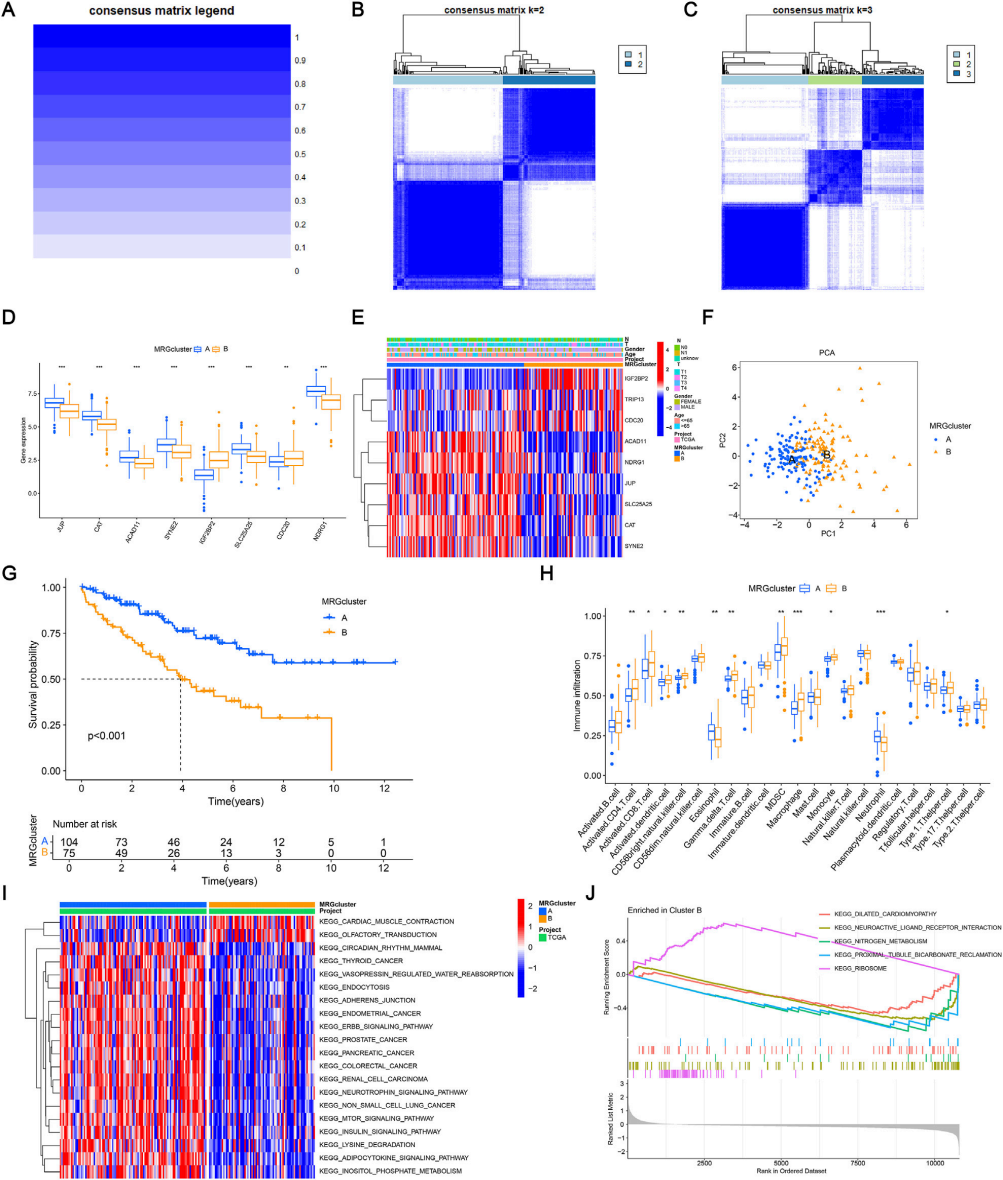
Analyzing immunological responses and consensus clustering. **(A–C)** Consensus clustering matrices for k = one to three based on the expression of nine MRGs, identifying two stable molecular subtypes. **(D)** Expression profiles of the nine MRGs in Cluster A and Cluster B. **(E)** Heatmap summarizing gene expression patterns and clinicopathological characteristics (gender, age, T stage, N stage) across the two clusters. **(F)** Principal component analysis (PCA) demonstrates a clear separation between the two clusters. **(G)** Kaplan-Meier survival curves comparing overall survival between Cluster A and Cluster B. **(H)** Boxplots showing differences in immune cell infiltration between the two clusters based on CIBERSORT estimates. **(I)** KEGG enrichment scores comparing pathway activity between the two clusters. Enrichment significance was determined using *FDR* < 0.05. **(J)** Gene Set Enrichment Analysis (GSEA) illustrates significantly enriched pathways in each cluster. GSEA was considered significant at *FDR* < 0.25, according to MSigDB recommendations.

A consensus clustering approach was used to conduct unsupervised clustering to find subgroups associated with mitophagy and explore possible clustering of ccRCC based on MRG expression. The nine MRGs were utilized to categorize ccRCC patients into distinct subgroups. The best classification parameter was k = 2 using the consensus clustering method, which divided ccRCC patients into two groups (Figures 2A–C). The expression levels of significant MRGs in clusters A and B are displayed in Figure 2D. Our comparative analysis reveals that these nine genes are strongly expressed in cluster A, further supported by the heat map in Figure 2E. The PCA plot shows a significant classification between classes A and B based on MRGs (Figure 2F). By using survival difference analysis, it was demonstrated that there was a substantial difference in the survival rates of the two cluster subgroups (*p* < 0.001), with cluster A having a higher survival rate (Figure 2G). The above results prove that clusters A and B can be dramatically distinguished according to the characteristics of these nine MRGs. When comparing immune cells in the two samples, the result revealed significant differences in most immune cells between the two clusters (Figure 2H). Our research demonstrated that immune cell infiltration can effectively differentiate subfamilies based on MRGs. Figure 2I shows the differences in KEGG pathway enrichment between paired clusters. The biochemical processes amongst various clusters differ, according to research. GSEA revealed that Cluster B was significantly enriched in several key pathways, including cell adhesion molecules, chemokine signaling pathways, cytokine-cytokine receptor interactions, hematopoietic cell lineage, and systemic lupus erythematosus. These findings suggest that Cluster B may play a critical role in immune regulation, cell communication, and inflammatory processes, providing valuable insights into the biological mechanisms associated with this MRG cluster (Figure 2J). These findings imply that MRGs might offer fresh perspectives on the immune system’s reaction and immunological infiltration in ccRCC.

### 3.4 Establishment and evaluation of mitophagy-related risk models

**FIGURE 3:**
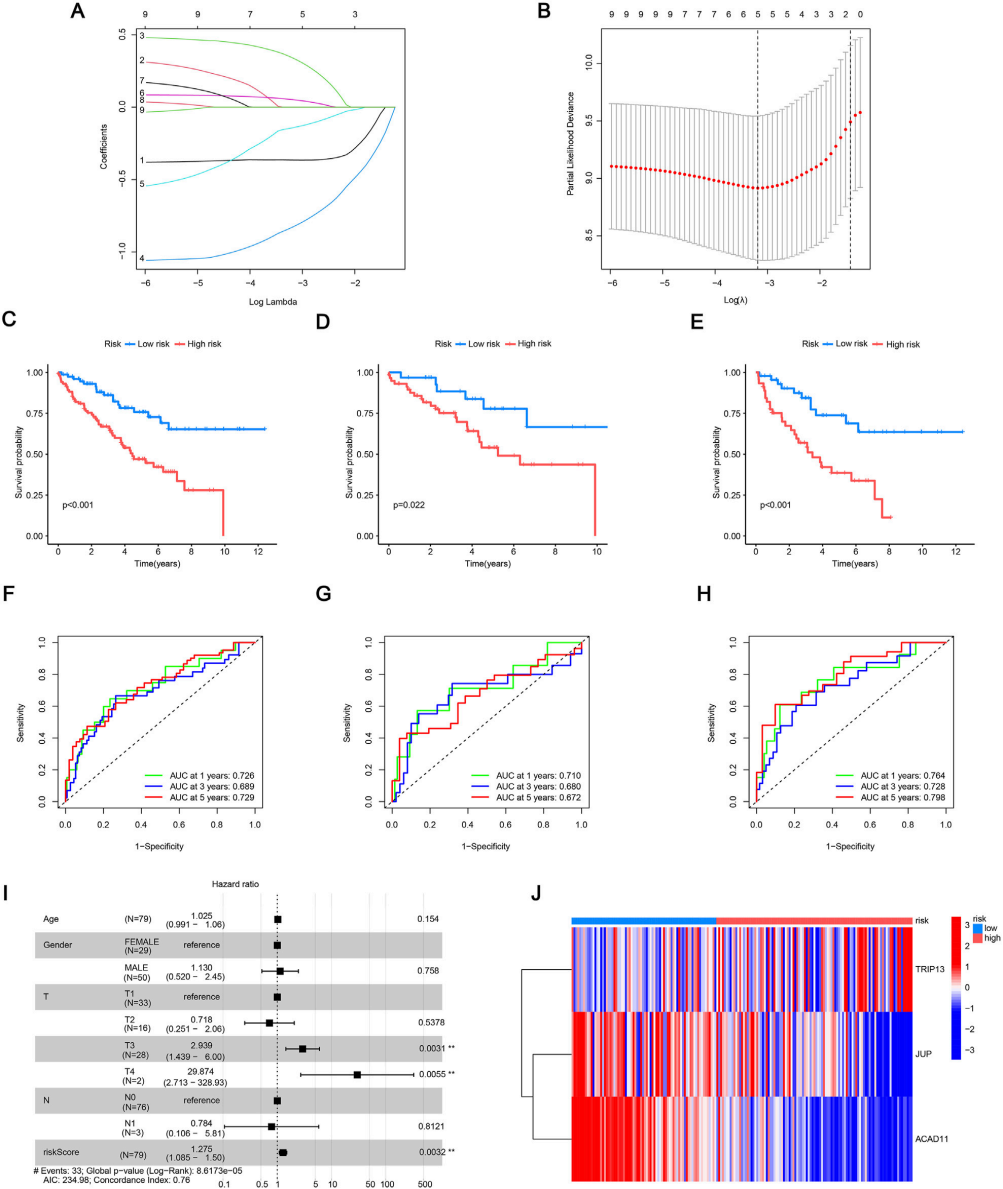
Construction and evaluation of the mitophagy-related risk model. **(A)** LASSO coefficient profiles of candidate MRGs. **(B)** Ten-fold cross-validation for selecting the optimal penalty parameter (lambda). **(C–E)** Kaplan-Meier survival curves comparing high- and low-risk groups in the entire cohort **(C)**, test cohort **(D)**, and training cohort **(E)**. **(F–H)** Time-dependent ROC curves evaluating predictive performance at 1, 3, and 5 years for the entire cohort **(F)**, test cohort **(G)**, and training cohort **(H)**. **(I)** Univariate Cox regression assessing the prognostic value of the risk score and clinical features. **(J)** Heatmap showing expression patterns of the three MRGs included in the risk model across high- and low-risk groups. *p < 0.05; **p < 0.01; and ***p < 0.001.

In order to measure the important influence of MRGs in patients with ccRCC, we built a risk model. This model enables the stratification of patients into high-risk and low-risk groups based on MRG expression profiles, providing a valuable tool for predicting prognosis and guiding personalized treatment strategies. Three core genes (JUP, TRIP13, and ACAD11) were identified in ccRCC, all of which were considered as prognostic indicators, and the risk model was constructed using these three genes (Figures 3A,B). The risk scores derived from multivariate analysis for each of the three screening MRGs are displayed in Supplementary Table S3. The following formula was used to determine the risk score: Risk score = (TRIP13 × 0.471404750400818) - (JUP × 0.439181231277887) - (ACAD11 × 1.21854543241319). Random assignments were made to the training and testing groups of ccRCC patients. Using K-M survival curves for both groups, we discovered that patients in the low-risk group fared better when it came to survival than patients in the high-risk group. This indicates that the model effectively distinguished the two risk categories (Figures 3C–E). We then used ROC curves to evaluate the degree of concordance between the forecasts made by the model and the actual data. Figure 3F shows the AUC values of all group at 1 year (0.726), 3 years (0.689), and 5 years (0.729) after surgery; testing group AUC values at 1 year (0.710), 3 years (0.680), and 5 years (0.672), respectively (Figure 3G); and training group AUC values at 1 year (0.764), 3 years (0.728), and 5 years (0.798), respectively (Figure 3H). The results of the study showed that the model has significant value in predicting patient survival. In addition, we also analyzed the changes in the survival status of ccRCC patients with risk scores. Our results show that as the risk score increases, the risk of death of patients also increases, and the number of deaths also increases. The same results are shown in both the test group and the train group (Supplementary Figure S3A–C). To independently assess the predictive importance of risk scores and specific clinical features, the analysis used univariate Cox regression. The findings presented in Figure 3I highlight that T3 and T4 stages and riskScore are key factors with regard to independent prognosis (*p* < 0.001). This combination of mitophagy-related risk scores and traditional TNM staging allows risk scoring to supplement traditional staging systems, helping to more accurately classify patient prognostic risks and providing a more personalized basis for clinical decision-making. The risk heatmap indicates that TRIP13 poses a high risk, whereas ACAD11 and JUP are identified as low-risk genes (Figure 3J). These findings indicate that the model underscores the critical role of MRGs in ccRCC progression and highlights their potential as therapeutic targets.

### 3.5 Construction and validation of the prognostic nomogram

**FIGURE 4:**
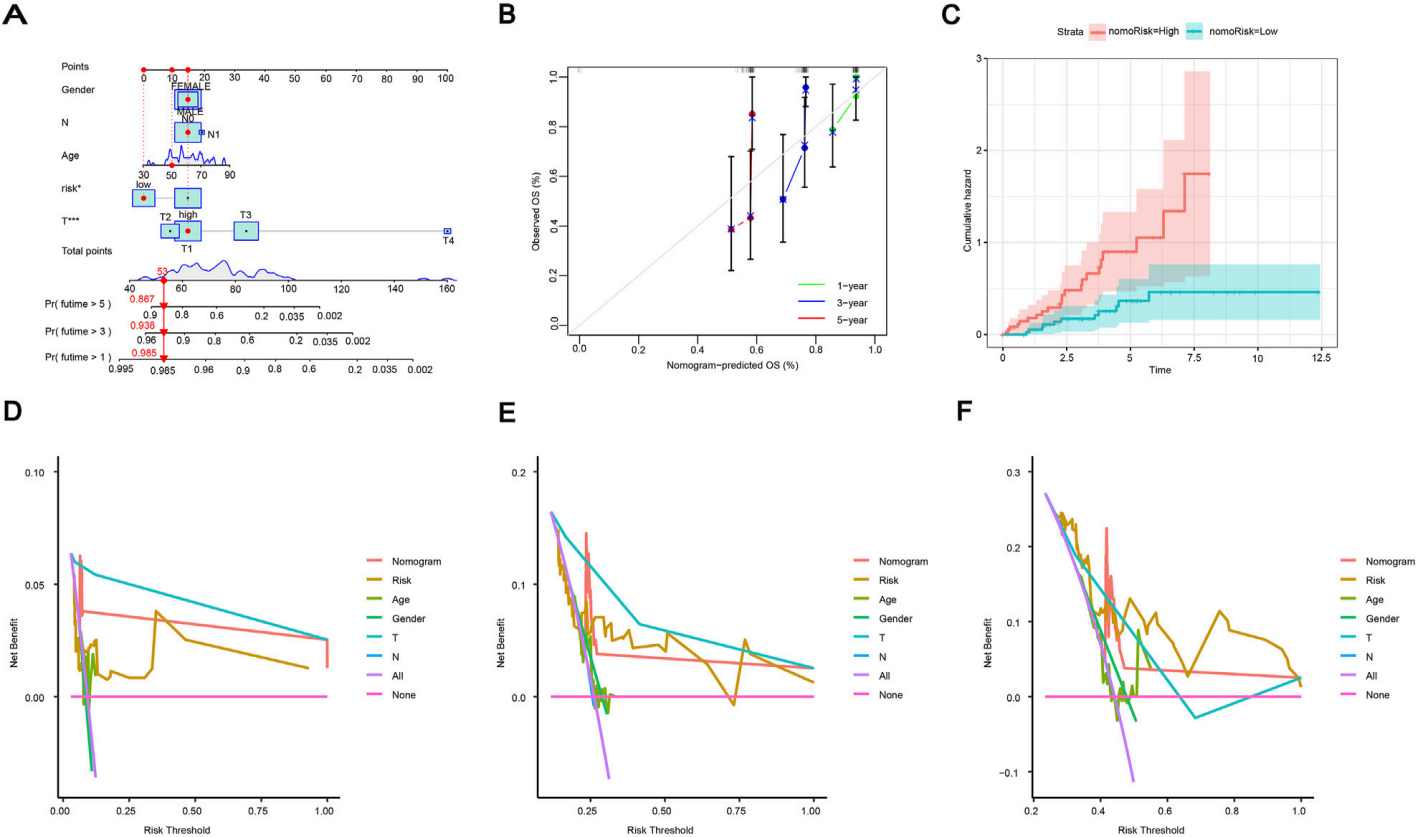
Construction and validation of the prognostic nomogram. **(A)** Nomogram integrating risk score and clinical characteristics to predict 1-, 3-, and 5-year overall survival in ccRCC. **(B)** Calibration curves showing the agreement between predicted and observed survival outcomes. **(C)** Nelson-Aalen cumulative hazard curves for the nomogram-derived risk groups. **(D–F)** Decision curve analysis (DCA) evaluating the clinical utility of the nomogram, risk score, and conventional clinical variables at 1 **(D)**, 3 **(E)**, and 5 years **(F)**. *p < 0.05; **p < 0.01; and ***p < 0.001.

To predict the survival of ccRCC patients, we created a nomogram and calibration curve. As shown in Figure 4A, the patient’s overall score was 53 points, indicating that the patient’s 1-, 3-, and 5-year survival rates were 0.985, 0.938, and 0.867, respectively. The calibration curve further verified that the 1–5 years survival rate of the patient was in line with the ideal state (Figure 4B). Over time, patients tend to become more susceptible to risk, especially those in the high-risk group, who are more susceptible than individuals in the low-risk category (Figure 4C). The decision curve shows that the developed risk model performs better than other clinical characteristics in predicting patient survival (Figures 4D–F). The above results indicate that the risk model we constructed can predict the overall survival rate of ccRCC patients to a certain extent.

### 3.6 Immune-related characteristics associated with the risk model

**FIGURE 5:**
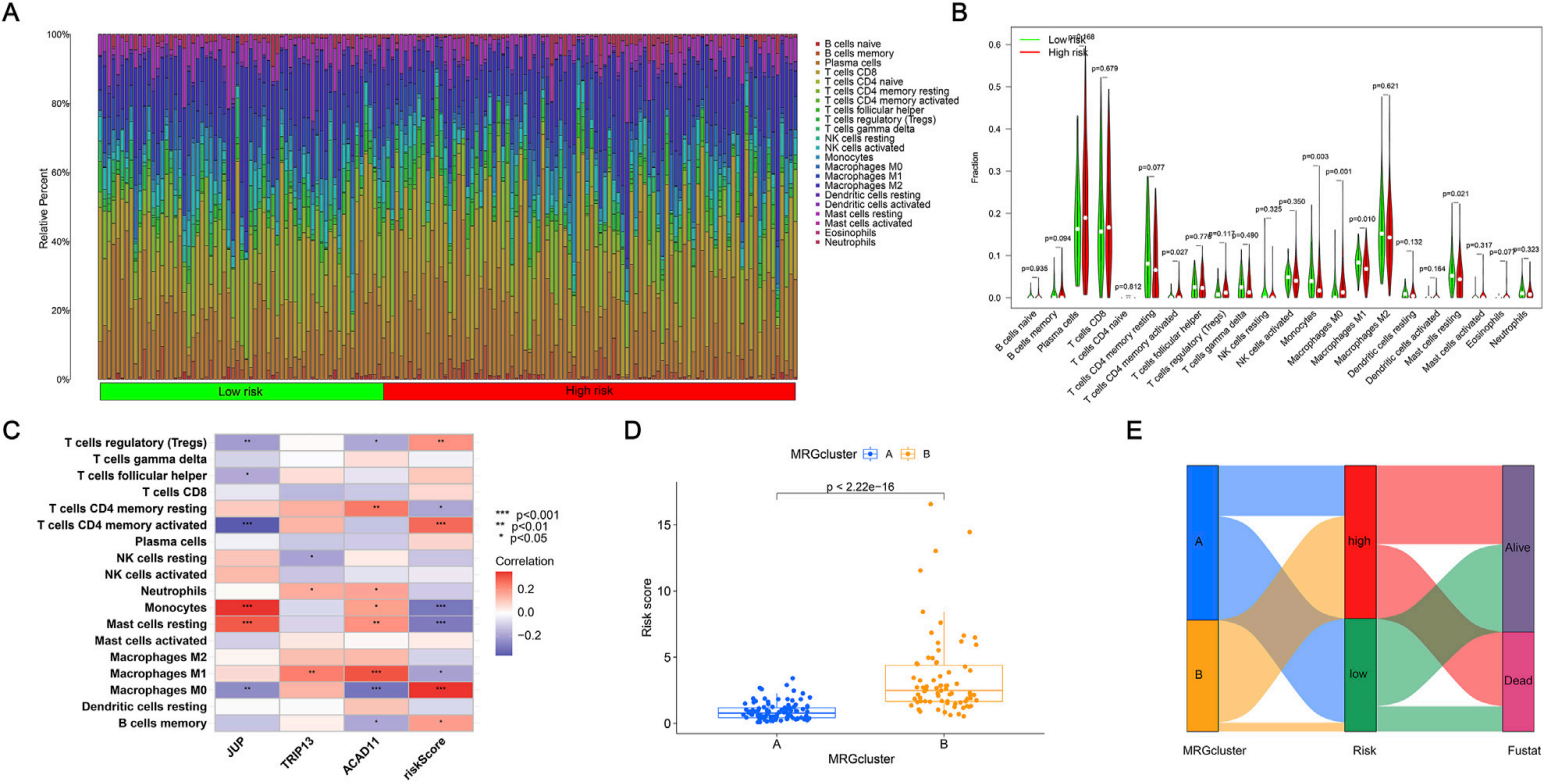
Immune-related characteristics associated with the risk model. **(A)** Immune cell infiltration profiles for individual patients in high- and low-risk groups. **(B)** Violin plots comparing the abundance of selected immune cell types between risk groups. **(C)** Heatmap illustrates correlations between immune cell populations and the three model-derived MRGs. **(D)** Correlation between risk scores and MRG-based molecular subtypes. **(E)** Sankey diagram showing associations among MRG clusters, risk groups, and patient survival status. *p < 0.05; **p < 0.01; and ***p < 0.001.

To investigate the association between mitophagy-related signatures and the tumor immune microenvironment, the CIBERSORT algorithm was applied to estimate the relative abundance of 22 immune cell types in high- and low-risk groups. The analysis revealed marked differences in immune cell infiltration patterns between the two groups, indicating that the risk score is closely associated with immune heterogeneity in ccRCC (Figure 5A). A correlation heatmap of immune cell subsets is provided in Supplementary Figure S4, illustrating the interactions among distinct immune populations. Further comparison of immune infiltration levels (Supplementary Figure S5) demonstrated significant variations in multiple immune cell types, including memory B cells, M0 macrophages, M1 macrophages, resting mast cells, monocytes, activated CD4^+^ memory T cells, resting CD4^+^ memory T cells, and regulatory T cells (Tregs). These findings suggest that the MRG-based risk signature reflects distinct immune microenvironmental states that may influence disease progression and therapeutic responsiveness. Five immune cell subsets exhibited particularly significant differences between high- and low-risk groups (Figure 5B). Correlation analysis between immune cells and the three model-derived MRGs revealed that ACAD11 showed the strongest association with immune infiltration patterns (Figure 5C), suggesting a potential role in shaping the immune microenvironment. We further examined the interaction between molecular subtypes and risk groups. Risk scores differed significantly between Cluster A and Cluster B (Figure 5D). The Sankey diagram (Figure 5E) demonstrated the correspondence among MRG-defined subtypes, risk categories, and clinical outcomes. In Cluster A, patients were distributed relatively evenly between high- and low-risk groups. In contrast, Cluster B contained a disproportionately higher percentage of high-risk patients, although most individuals in this cluster exhibited favorable survival outcomes. Together, these results highlight the distinct immune characteristics and risk profiles associated with MRG-based classifications, providing additional insight into their prognostic implications in ccRCC.

### 3.7 Predicted drug sensitivity between high- and low-risk groups

**FIGURE 6:**
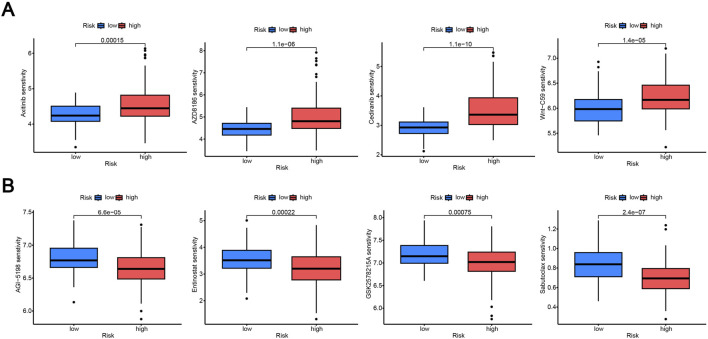
Predicted drug sensitivity between high- and low-risk groups. **(A)** Representative drugs predicted to have greater sensitivity (lower IC50 values) in the high-risk group. **(B)** Representative drugs predicted to have greater sensitivity in the low-risk group. Drug sensitivity was inferred using IC50 predictions generated by the oncoPredict algorithm based on GDSC data.

In clinical practice, ccRCC patients are often treated with chemotherapy, radiotherapy, or targeted therapy, many of which are associated with significant side effects. As a result, drug therapy plays a crucial role in the management of ccRCC. Through drug sensitivity analysis, we identified a total of 60 drugs with different sensitivities between the two groups. Figure 6A shows four drugs that are much more sensitive in the high-risk group than in the low-risk group, indicating that the IC50 values of these drugs in the high-risk group are significantly lower than those in the low-risk group, indicating that these drugs are more effective for patients in the high-risk group. Figure 6B shows four drugs that are more sensitive in the low-risk group than in the high-risk group, indicating that the IC50 values of these drugs in the low-risk group are significantly lower than those in the high-risk group, further indicating that these drugs are more effective for low-risk patients. The sensitivity of the remaining drugs in different groups is shown in Supplementary Table S4. By analyzing the differences in IC50 of chemotherapy drugs in high-risk and low-risk groups, it can help doctors optimize treatment plans for patients and provide potential basis for selecting appropriate therapeutic drugs and combination therapy. These findings suggest that risk stratification based on the MRG signature may inform potential therapeutic strategies; however, further experimental and clinical validation is required.

### 3.8 Dentification and preliminary validation of key prognostic genes

**FIGURE 7:**
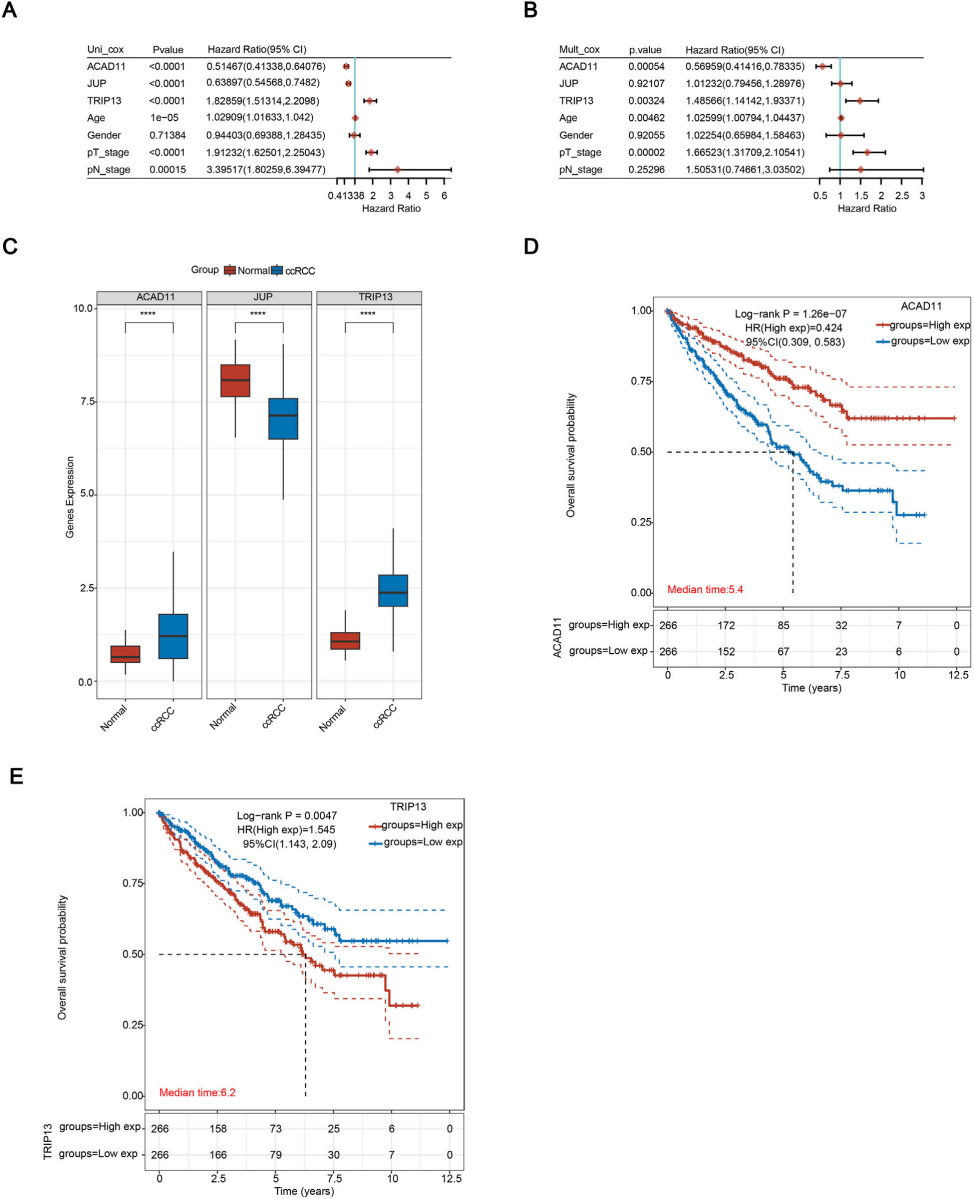
Dentification and preliminary validation of key prognostic genes. **(A,B)** Univariate and multivariate Cox regression analyses evaluating clinical characteristics and the three model-derived MRGs. **(C)** mRNA expression levels of the three genes in normal renal tissues and ccRCC tissues. **(D,E)** Kaplan-Meier survival curves for ACAD11 **(D)** and TRIP13 **(E)** stratified by expression level. *p < 0.05; **p < 0.01; ***p < 0.001; and ****p < 0.0001.

To further screen independent predictive markers for ccRCC, we performed univariate and multivariate Cox analysis and found that age, pT stage, TRIP13, and ACAD11 were independent predictors (Figures 7A,B). Compared with nearby normal tissues, the expression levels of ACAD11 and TRIP13 were significantly upregulated in ccRCC tissues (Figure 7C). However, from Figures 7D,E, we found that only the survival rate of TRIP13 in the high expression group was significantly lower than that of the low expression group, while the survival rate of the ACAD11 high expression group was higher than that of the low expression group, indicating that TRIP13 has a significant function in ccRCC and can serve as an independent prognostic gene in the disease. By drawing a circle diagram, the gene in the inner circle is TRIP13, and the genes in the outer circle are predicted to have similar functions or common characteristic genes with TRIP13. The interactions and functions between these genes are shown in Supplementary Figure S6. It can be seen that the main function of TRIP13 is related to the cell cycle and mainly mediates the occurrence and development of tumors.

### 3.9 Verify TRIP13 expression and investigate its predictive role in ccRCC

**FIGURE 8:**
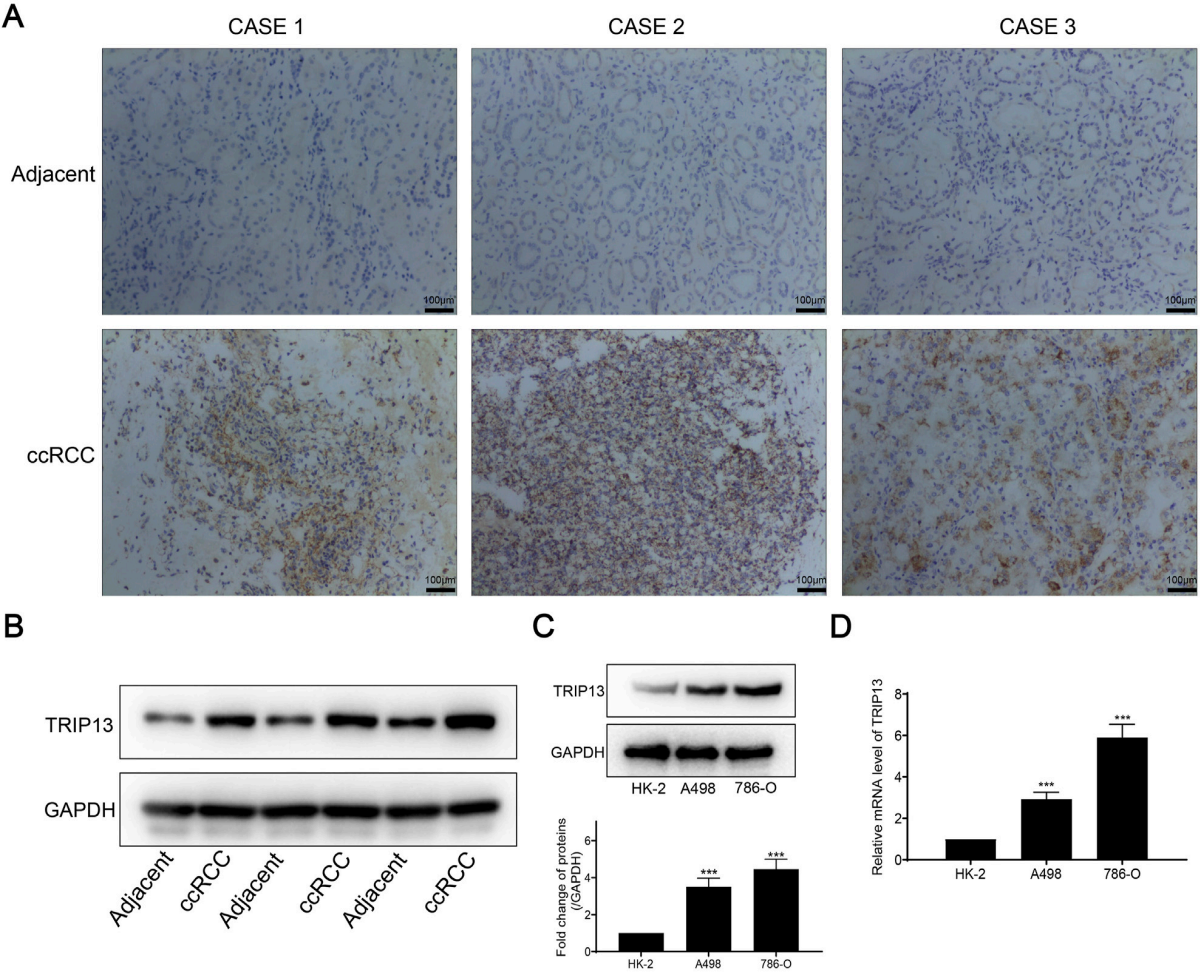
Expression of TRIP13 in ccRCC tissues and cell lines. **(A)** Immunohistochemistry was used to assess TRIP13 expression in ccRCC and surrounding tissues (n = 3). The scale is 100 μm. **(B)** Western blotting was performed to detect TRIP13 (46 kDa) expression levels in ccRCC tumor tissues and paired adjacent normal tissues (n = 3). **(C)** The expression of TRIP13 was assessed using the Western blot method in two ccRCC cell lines and one normal kidney cell line (n = 3, ***p < 0.001). **(D)** The expression of TRIP13 was examined by PCR in one normal renal cell line and two ccRCC cell lines (n = 3, ***p < 0.001).

**FIGURE 9:**
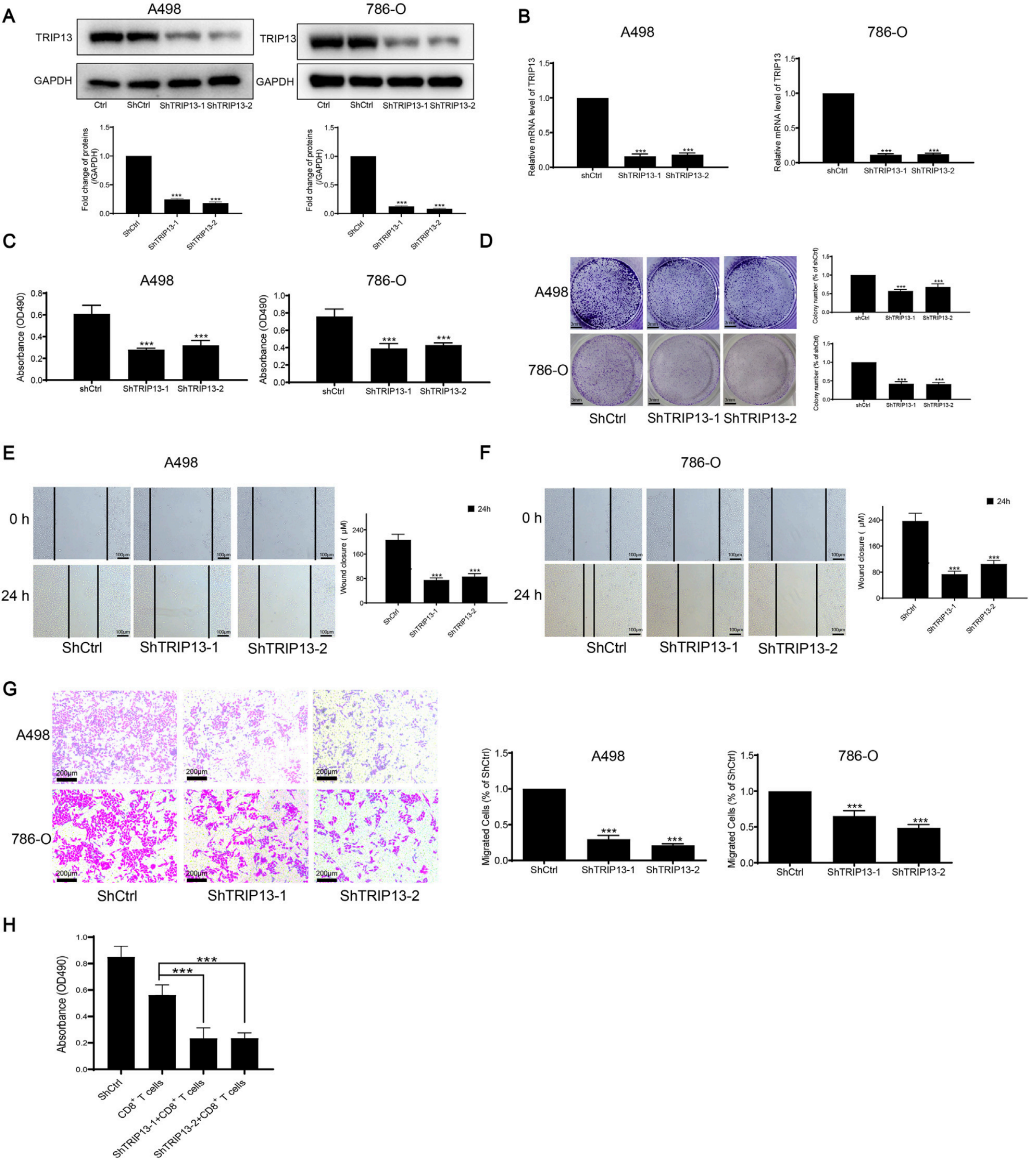
Regarding the validation of the prognostic function of TRIP13 in ccRCC. **(A)** Following the silencing process, Western blot analysis was performed to assess TRIP13 (46 kDa) expression (n = 3, ***p < 0.001). **(B)** After silencing TRIP13, we utilized PCR to measure the expression of TRIP13 (n = 3, ***p < 0.001). **(C)** After TRIP13 was silenced, cell proliferation was detected by MTT assay (n = 3, ***p < 0.001). **(D)** A colony formation assay was used to measure the dramatic suppression of colony formation after TRIP13 knockdown (n = 3, ***p < 0.001). **(E–G)** After TRIP13 was silenced, the effect of migration suppression was assessed using the scratch and transwell assay (n = 3, ***p < 0.001). **(H)** Explore the cell survival of shCtrl and shTRIP13 tumor cells when treated with CD8^+^ T cells (n = 3, ***p < 0.001).

In our investigation of TRIP13 expressions in ccRCC, we conducted TRIP13 staining on three adjacent cancer tissues and three ccRCC tissues. TRIP13 was shown to be substantially more expressed in ccRCC tissues than in neighboring tissues, according to the WB data (Figure 8A). Next, we used WB and PCR to look at TRIP13 expression in two ccRCC cell types and one normal kidney cell type. According to Figures 8B–D, when ccRCC was compared to normal cells, TRIP13 had higher protein and mRNA levels. This led us to delve deeper into TRIP13’s role in ccRCC. PCR and WB were used to confirm that TRIP13 had been successfully silenced (Figures 9A,B). We then assessed the impact of TRIP13 on ccRCC using MTT, clonogenic, scratch, and transwell assays. The results showed that there was a significant reduction in cell migration, clonogenesis, and proliferation when TRIP13 was silenced (Figures 9C–G). Notably, in the absence of TRIP13, human ccRCC cells were more susceptible to destruction by activated T cells during co-incubation with CD8^+^ T cells (Figure 9H). The crucial role of TRIP13 in immune evasion, metastasis, and proliferation of ccRCC cells is highlighted by these findings.

### 3.10 *In vivo* ccRCC cell growth and migration were suppressed by TRIP13 knockdown

**FIGURE 10:**
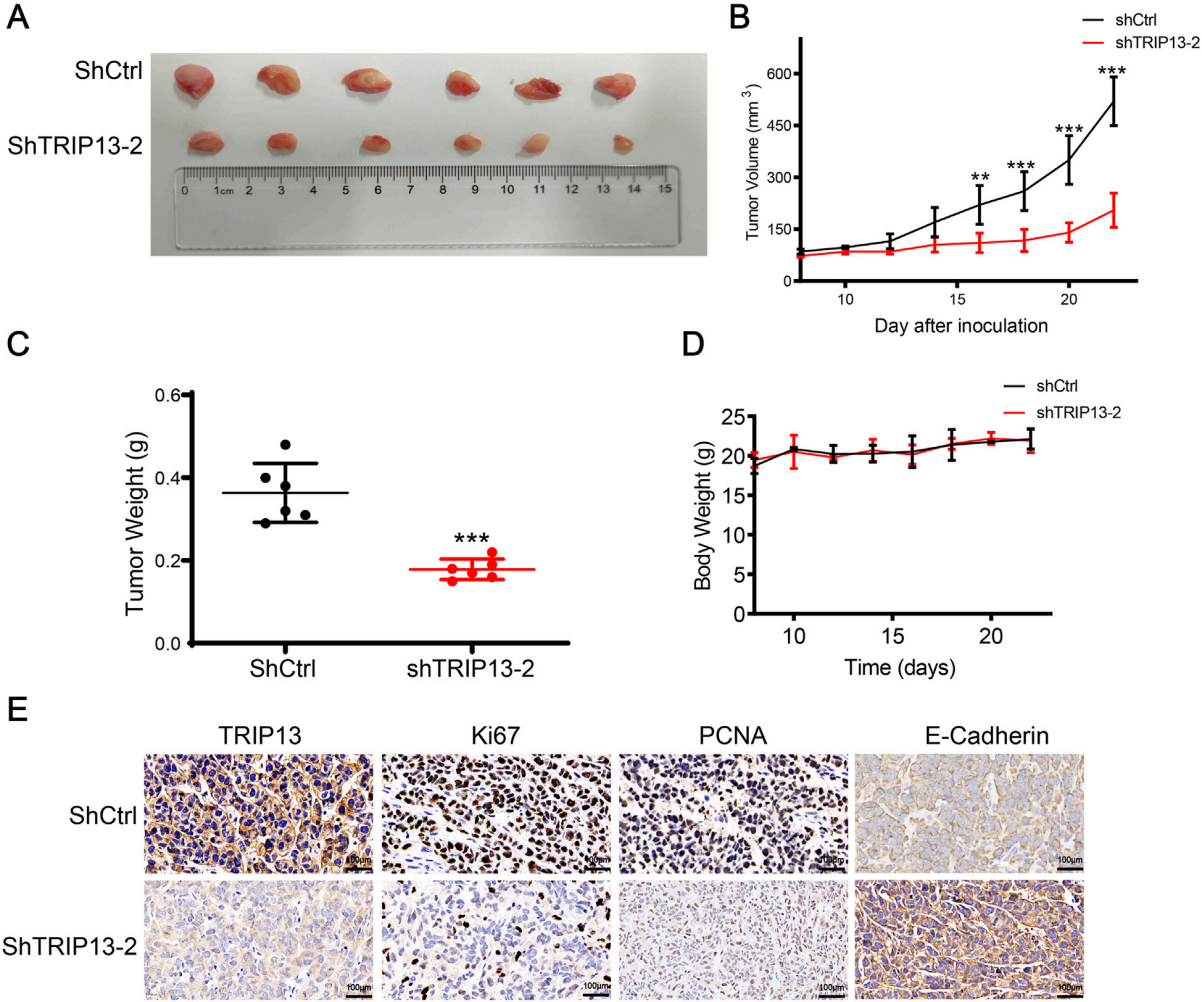
Effects of TRIP13 knockdown on tumor growth *in vivo*. **(A,B)** The mice were subcutaneously injected with a suspension of 2 × 10^6^ A498-shCtrl or A498-shTRIP13 cells into their right flank. Tumor growth was monitored starting 8 days post-injection, when the tumor volume reached 70–100 mm^3^, and measurements were taken every 2 days thereafter. On day 22 after cell implantation, the lumbar tumors were excised and photographed for further analysis (n = 6, ***p < 0.001). **(C)** The tumor weight was recorded 22 days after the operation (n = 6, ***p < 0.001). **(D)** Each group’s mice’s body weight was altered (n = 6). **(E)** Immunohistochemistry was used to identify tumor samples' TRIP13, Ki67, PCNA and E-Cadherin expression(n = 3).

The effect of TRIP13 knockdown on tumor formation was examined using a subcutaneous xenograft model in nude mice. Following implantation of non-targeting control (shCtrl) and shTRIP13-2 expressing cells, tumor growth was suppressed in the TRIP13 knockdown group (Figures 10A,B). The tumor weight in the shTRIP13-2 group was 53% lower than that in the shCtrl group (Figure 10C). The average body weight of the mice was not significantly affected by TRIP13 knockdown (Figure 10D). Immunohistochemical analysis showed that the positive staining intensity of proliferation-related proteins Ki-67 and PCNA was significantly lower in the shTRIP13-2 group than in the shCtrl group, while the positive staining intensity of migration-related protein E-cadherin was significantly higher in the shCtrl group (Figure 10E). These results indicate that TRIP13 knockdown inhibits the expression of PCNA and Ki-67 and enhances the expression of E-cadherin, thereby inhibiting the proliferation and migration of ccRCC cells *in vivo*.

## 4 Discussion

Aberrant mitophagy in ccRCC may be induced by gene mutations, abnormal regulatory system function, and changes in the extracellular and intracellular environment (Sun et al., 2022). In ccRCC tumor tissues, renal clear cells demonstrated significantly elevated levels of mitophagy. Additionally, a correlation was observed between high expression of key genes associated with increased mitophagy and poor prognosis in patients (Jiang et al., 2024). Some studies have highlighted the significant impact of inducing mitophagy on reducing the proliferation and migration of ccRCC (Yan et al., 2023). These MRGs are closely associated with the development and progression of ccRCC, and growing evidence supports this theory. They may also serve as potential biomarkers with practical clinical applications.

Our study provides new insights into the role of mitophagy in ccRCC by constructing a prognostic model based on MRGs and conducting an immunological correlation analysis to identify potential prognostic biomarkers. To develop a predictive risk score model for ccRCC, we evaluated three genes (JUP, TRIP13, and ACAD11), some of which have been previously linked to tumorigenesis and progression. While these three genes were derived from the MRG set, the present findings mainly reflect their prognostic value rather than direct functional involvement in mitophagy. As an important member of the catenin family, JUP (Junction Plakoglobin) maintains cell-cell adhesion and structural integrity by connecting cadherin to the actin cytoskeleton (Li et al., 2011). Its downregulation can destroy cell-cell junctions and significantly enhance tumor invasion and metastasis (Holen et al., 2012). In different cancers, JUP exhibits dual effects: in non-small cell lung cancer (NSCLC), it plays a tumor suppressor function by inhibiting the β-catenin/TCF signaling pathway (Winn et al., 2002); while in malignant tumors such as breast cancer and leukemia, its high expression is associated with poor prognosis (Morgan et al., 2013; Goto et al., 2017). It is worth noting that in ccRCC, JUP shows characteristic low expression, and functional experiments have confirmed that its knockdown promotes tumor occurrence and development, while overexpression significantly inhibits the malignant phenotype of tumors (Chen et al., 2021). Clinical analysis further showed that low expression of JUP in ccRCC patients was significantly associated with poor prognosis, suggesting that JUP may serve as a new therapeutic target for ccRCC. ACAD11-mediated fatty acid oxidation (FAO) is a key metabolic pathway for cells to maintain energy homeostasis when glucose is deficient (Hanahan and Weinberg, 2011). p53 tumor suppressor reverses tumor metabolic reprogramming and inhibits tumor progression by upregulating ACAD11, inhibiting the Warburg effect (aerobic glycolysis), and promoting oxidative phosphorylation (OXPHOS) (Schwartzenberg-Bar-Yoseph et al., 2004). In ccRCC, ACAD11 was identified as an important prognosis-related gene, and its expression level was closely related to the infiltration of various immune cells in the tumor microenvironment. This finding reveals that ACAD11 may play a key role in the progression of ccRCC through a dual mechanism - regulating tumor metabolic reprogramming and affecting tumor-immune microenvironment interactions, making it a highly potent target for therapeutic intervention.

Notably, TRIP13 was identified as an independent prognostic gene with significant clinical value. TRIP13 is a conserved AAA + ATPase that maintains genomic stability by regulating chromosome segregation and DNA repair. Aberrant activation or overexpression of TRIP13 can induce chromosomal instability (CIN) and promote tumorigenesis (Marks et al., 2017; Yost et al., 2017). As a key regulator of the spindle assembly checkpoint (SAC), TRIP13 catalyzes the conversion of active MAD2 to its inactive form, thereby facilitating mitotic progression (Firestone et al., 2012). Additionally, TRIP13 has been reported to favor error-prone non-homologous end joining (NHEJ) during double-strand break (DSB) repair, further exacerbating genomic instability. Similar to PARP-mediated repair dependence, this feature suggests its potential as a therapeutic target, particularly in homologous recombination-deficient tumors (Brown et al., 2017; Banerjee et al., 2014). TRIP13 overexpression is also associated with chemotherapy resistance, promotes the progression of head and neck cancer, breast cancer, and colorectal cancer, and is significantly associated with poor prognosis in patients (Banerjee et al., 2014; Wang et al., 2014; Sheng et al., 2018). Functionally, TRIP13 promotes breast cancer cell proliferation and migration, supporting its role as a driver of tumor progression and a potential diagnostic or therapeutic target (Lan et al., 2022). Consistent with these studies, our work identified TRIP13 as an independent prognostic biomarker in ccRCC and incorporated it into a clinically meaningful risk model. Functional experiments confirmed that TRIP13 contributes to ccRCC malignancy by enhancing tumor proliferation (via Ki67 and PCNA), promoting migration and invasion, and facilitating immune escape. *In vivo* xenograft assays further validated that TRIP13 knockdown markedly suppresses tumor growth, emphasizing its role as a multifaceted oncogenic driver. Although these findings demonstrate TRIP13’s tumor-promoting effects, its mechanistic relationship with mitophagy remains largely unclear. TRIP13 has been linked to ATPase-dependent remodeling and metabolic stress adaptation, but direct evidence showing that TRIP13 regulates mitophagy is lacking. In particular, whether TRIP13 modulates canonical mitophagy pathways—such as PINK1/Parkin-mediated ubiquitination (Li et al., 2023), LC3-II conversion (Ma et al., 2018), or BNIP3/BNIP3L-driven mitochondrial clearance (Chen et al., 2023)—has not been determined. Future studies examining mitophagy flux, mitochondrial membrane potential, mtROS levels, and autophagy marker dynamics will be essential to clarify whether TRIP13 influences mitochondrial quality control in ccRCC or acts through alternative metabolic or signaling pathways. Studies have found that TRIP13 is an indicator of poor prognosis in ccRCC, and the higher its expression, the worse the prognosis of ccRCC (Chen X. et al., 2025). This is consistent with our results. In order to find the upstream mRNA of TRIP13, we screened the target miRNA of TRIP13 through four online predicted miRNA databases: miRTarBaseV8.0, StarBase3.0, miRDB and miRWalk. Similar analytical methods have been applied to other types of cancer research. Studies have shown that serum exosomes hsa-let-7f-5p can serve as potential biomarkers for the detection and diagnosis of metastatic pancreatic cancer using this method (Ren et al., 2025). Therefore, our study also utilized this method to further identify TRIP13-related miRNAs to explore the role of TRIP13 in ccRCC. We found that hsa-miR-92b-3p overlapped (Supplementary Figure S7). Some studies have found that hsa-miR-92b-3p is involved in acute kidney injury (Liu et al., 2025), but the impact on ccRCC has not been reported. We speculate that the biological function of ccRCC may be affected by hsa-miR-92b-3p/TRIP13. In addition, our KEGG enrichment showed that the HIF-1 signaling pathway was active. HIF-1 is a key regulatory factor in ccRCC. It is derived from VHL inactivation. It promotes lipid accumulation in ccRCC by inhibiting fatty acid metabolism, thereby promoting the occurrence and development of cancer (Du et al., 2017). However, the specific mechanism of TRIP13 in ccRCC still needs to be further verified, especially whether it indirectly affects immune checkpoint molecules by regulating mitochondrial homeostasis, thereby affecting the progression of ccRCC. Therefore, in future studies, we will also focus on studying the mechanism of TRIP13 affecting ccRCC and immune escape, etc., to promote personalized treatment of ccRCC patients.

In addition to the prognostic significance of MRG-based biomarkers, our study also identified several pathways closely related to the pathogenesis of ccRCC, such as the HIF-1 signaling pathway, the PPAR signaling pathway, and glycogen metabolism-related pathways. These pathways warrant further investigation because they are closely related to mitochondrial homeostasis and mitophagy. Due to the deletion of the VHL gene, the HIF-1 signaling pathway becomes a crucial factor in the development of ccRCC (Chen et al., 2016). This deletion triggers a series of metabolic changes, including enhanced glycolysis, lipid accumulation, and abnormal oxygen-sensing mechanisms. Recent research indicates that HIF-1α activation can regulate mitophagy through BNIP3/BNIP3L-mediated mitochondrial clearance (Fu et al., 2020), suggesting that hypoxia-induced transcriptional changes may indirectly affect mitophagy activity in ccRCC. Similarly, the PPAR signaling pathway plays a key role in lipid metabolism, fatty acid oxidation, and mitochondrial formation (Zou et al., 2024; Li et al., 2020; Haemmerle et al., 2011). Dysregulation of PPARα and PPARγ leads to decreased mitochondrial quality control and abnormal mitophagy (Zhang et al., 2022), potentially increasing the metabolic risk in ccRCC patients. Abnormalities in glycogen metabolism-related pathways are consistent with the metabolic characteristics of ccRCC, as abnormal glucose utilization and glycogen storage are frequently observed in ccRCC patients (Ochocki et al., 2018). Studies have shown that mitochondrial dysfunction and mitophagy directly affect the efficiency of glycolysis and the rate of glycogen metabolism, further illustrating the close relationship between energy metabolism and mitochondrial quality control (Tang et al., 2023). In summary, these pathways related to the pathogenesis of ccRCC suggest that MRG-based risk stratification may reflect multiple metabolic abnormalities associated with mitophagy regulation. Although current analyses only show correlations rather than direct causal relationships, these findings provide a biological explanation for how mitophagy-related genes affect the metabolic and immune status of ccRCC.

Immunotherapy represents a paradigm shift in cancer treatment by augmenting the immune system’s ability to recognize and eliminate malignant cells (Xu et al., 2024; Wang et al., 2025; Riley et al., 2019; Sensi et al., 2024). A cornerstone of this approach involves activating T-cell responses against tumor-specific antigens, which has demonstrated remarkable clinical efficacy across multiple cancer types (Kumar et al., 2021). Immune infiltration, a significant part of the tumor microenvironment (TME), has contributed to tumor progression and immunotherapy response (Balkwill et al., 2012). The efficacy of cancer immunotherapy has shown that tumor cells can be eliminated by immune cells, especially T lymphocytes (Darvin et al., 2018). Through innovative integration of mass spectrometry and high-dimensional flow cytometry, Chevrier et al. (2017) revolutionized our understanding of the TME in ccRCC. Their seminal work provided the first comprehensive single-cell atlas of immune infiltration in ccRCC, revealing previously unappreciated heterogeneity in immune cell phenotypes and functional states within the TME. This systematic profiling identified novel immunosuppressive cellular networks that contribute to ccRCC progression and therapy resistance (Chevrier et al., 2017). The TME represents a highly dynamic ecosystem composed of malignant cells, immune populations (T cells, macrophages, dendritic cells), stromal components (fibroblasts, endothelial cells), and extracellular matrix. This complex cellular network engages in continuous bidirectional crosstalk through cytokine signaling, cell-cell contacts, and metabolic competition, collectively shaping an immunosuppressive niche that promotes tumor progression, metastasis, and therapeutic resistance (Zhang and Zhang, 2020; Zinovkina et al., 2024). Our study established a novel MRG-based risk score model that showed significant associations with distinct immune cell infiltration patterns in ccRCC. These findings suggest that metabolic reprogramming signatures may reflect differences in the tumor immune microenvironment, potentially providing clues to mechanisms underlying immunotherapy resistance.

In addition, we screened 60 anticancer drugs through drug sensitivity analysis and found that there was a strong relationship between risk and score, indicating that these anticancer drugs have potential value in the treatment of ccRCC with different risks. Currently, the treatment drugs for RCC include targeted therapy, immunotherapy, and chemotherapy (Yamana et al., 2022). Although these drugs are effective, they often bring adverse effects to patients due to the toxic side effects of the drugs and the limited therapeutic effect. Therefore, it is beneficial to improve patient satisfaction by formulating individualized treatment plans according to the different conditions of patients. Our study found that most drugs are more sensitive to patients in the high-risk group, indicating that these drugs will achieve more satisfactory results in the treatment of high-risk patients. However, the treatment of low-risk patients not only fails to achieve the purpose of treatment but may also aggravate the symptoms of patients. In addition, eight drugs are more sensitive in low-risk patients, indicating that these drugs are effective in the treatment of low-risk patients. Entinostat is a synthetic benzamide derivative class I histone deacetylase (HDAC) inhibitor that can inhibit cell proliferation and promote breast cancer cell apoptosis, thereby treating breast cancer (Trapani et al., 2017). Studies have also found that entinostat can be used for immunomodulation of renal cell RCC treated with high-dose interleukin-2 to enhance the therapeutic effect of RCC (Pili et al., 2017). This shows that entinostat has great value in the treatment of RCC, and whether it can be used as a treatment for low-risk ccRCC patients requires more clinical trials. Although our study predicted 60 potential drugs, these drug sensitivity findings are based solely on *in silico* IC50 prediction derived from GDSC cell line datasets and should not be interpreted as evidence of actual clinical efficacy. The results reflect potential drug sensitivity patterns that require further preclinical and clinical validation.

While our prognostic model of MRGs provides potential clinical insights for ccRCC management, several limitations must be acknowledged. First, the reliance on publicly sourced datasets may have resulted in incomplete clinical annotations, batch effects, and unknown confounders that could not be fully eliminated despite normalization procedures. Second, the risk model prioritized a select set of mitophagy-related genes based on bioinformatic filtering, which may overlook additional biologically relevant regulators involved in mitochondrial quality control, immune modulation, or ccRCC progression. Third, although our experimental verification confirms the functional role of TRIP13 in ccRCC, the number of clinical tissue samples and animal experiments in this study remains limited, which may reduce the robustness and generalizability of the findings. Larger, multi-center cohorts and more extensive *in vivo* studies are required to substantiate the prognostic and biological significance of TRIP13. Fourth, the current study lacks detailed mechanistic evidence regarding how TRIP13 modulates ccRCC development, immune evasion, or mitochondrial homeostasis. Our data do not clarify whether TRIP13 directly regulates mitophagy or influences tumor progression through alternative pathways such as DNA repair, metabolic reprogramming, or immune checkpoint modulation. Additional mechanistic studies, _such as mitochondrial functional assays, rescue experiments, and targeted pathway inhibition, are necessary to establish causal relationships. Finally, the predicted drug sensitivity results were derived solely from *in silico* IC50 estimates based on the GDSC database and may not accurately reflect real-world clinical responses, as most predicted drugs lack validated evidence in RCC. Therefore, further experimental validation through *in vitro* drug-response assays, patient-derived organoids, or clinical datasets is required to confirm the translational relevance of these predictions. Taken together, these limitations highlight the need for future large-scale, mechanistic, and clinically integrated studies to strengthen the conclusions and facilitate the clinical application of MRG-based prognostic signatures in ccRCC.

## 5 Conclusion

Through comprehensive bioinformatics analysis, we demonstrated the importance of MRGs in ccRCC. The MRG-based risk model accurately predicts prognosis and immunological conditions in ccRCC patients. TRIP13, a significant independent prognostic gene, plays a pivotal role in ccRCC progression by modulating cell growth, migration, and invasion. These insights provide potential targets for developing innovative treatments for ccRCC patients.

## Data Availability

The original contributions presented in the study are included in the article/Supplementary Material, further inquiries can be directed to the corresponding authors.
