# Supplementary material for: Mitophagy-related gene TRIP13 predicts prognosis and immune response and promotes proliferation and migration in vitro and in vivo of clear cell renal cell carcinoma
Source: Front Pharmacol. 2025 Dec 18;16:1736086. doi: 10.3389/fphar.2025.1736086 (PMC12756432; doi:10.3389/fphar.2025.1736086)
Supplement: Supplementary file 1 [file Supplementaryfile1.docx]

Supplementary Material

**Supplementary Figures 1-7 and Supplemental Tables 1-4**

## Supplementary Figures 1-7

**Figure S1**


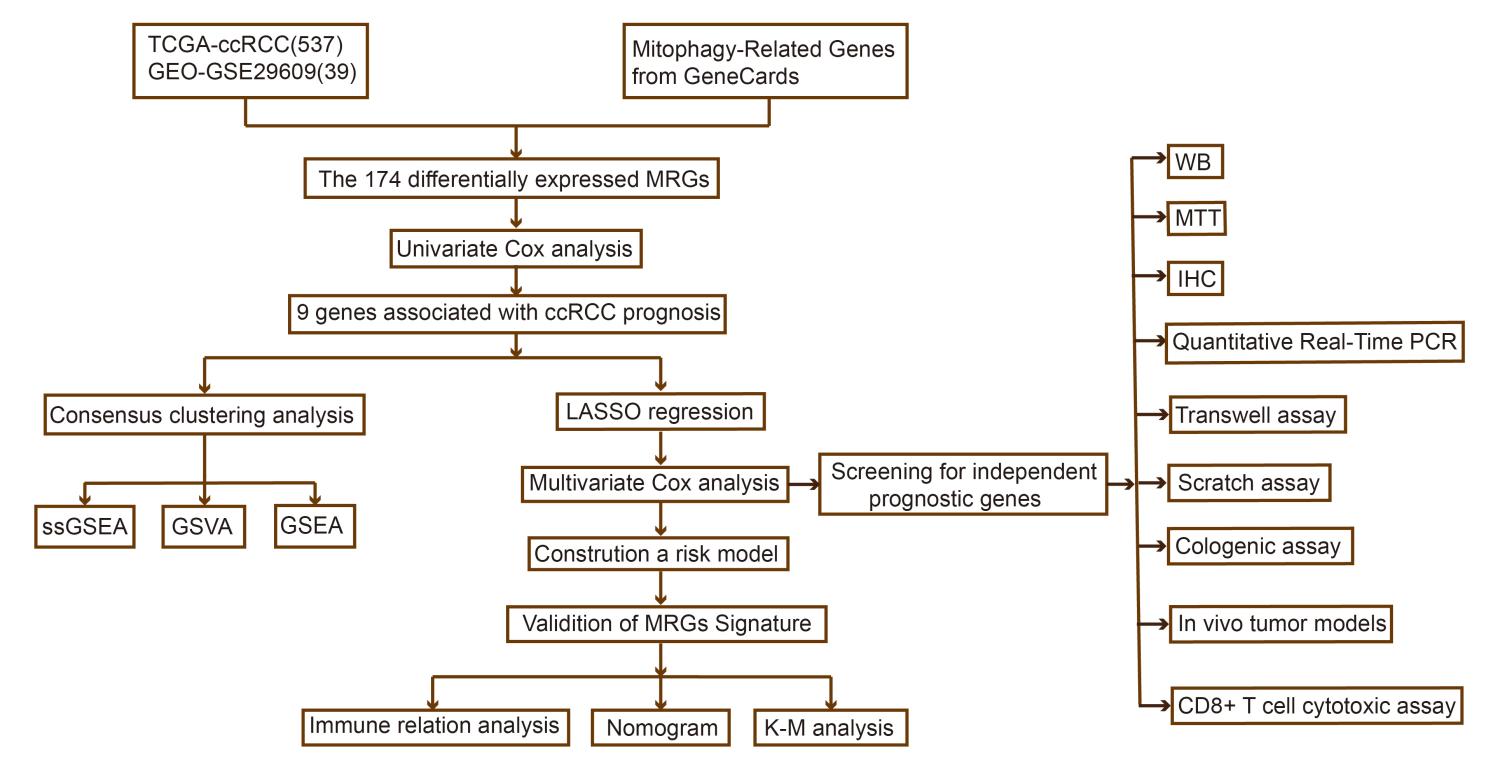


**Figure S1.** **Flow diagram for this research.**

TCGA, The Cancer Genome Atlas; GEO, the Gene Expression Omnibus; MRGs, mitophagy-related genes; ssGSEA, single sample Gene Set Enrichment Analysis; GSVA, Gene Set Variation Analysis; GSEA, Gene Set Enrichment Analysis; WB, Western blot; IHC, Immunohistochemical staining.

**Figure S2. Volcano plot of DEGs and GO and KEGG enrichment analysis.**


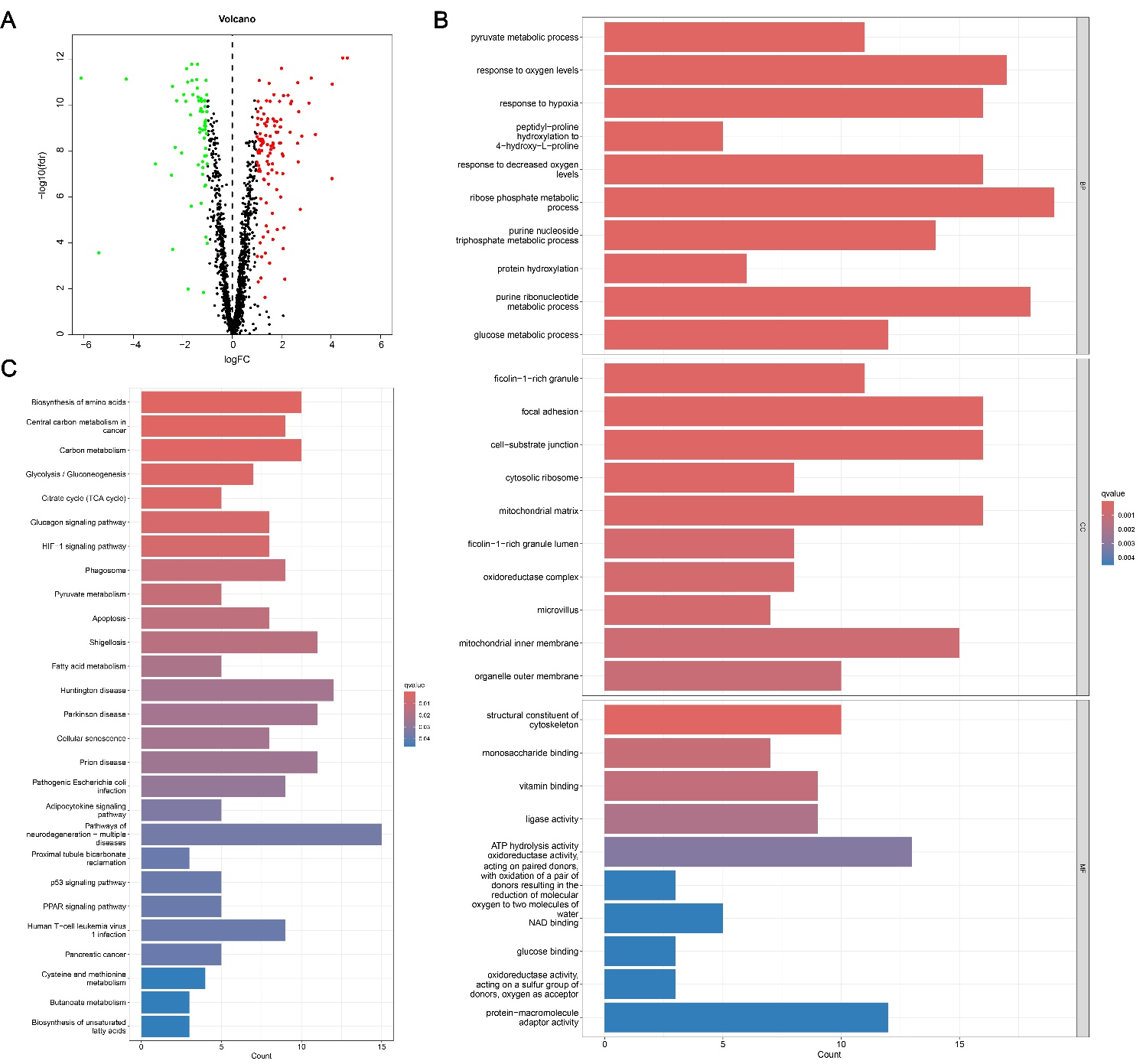


**Figure S2. Volcano plot of DEGs and GO and KEGG enrichment analysis.** (A) The volcano plot displaying the 174 differentially expressed mitophagy-related genes (DEGs) between ccRCC and normal tissues. The screening criteria for these genes are |logFC| ≥ 1 and *FDR* < 0.05.. Green represents down-regulated genes, red represents up-regulated genes, and black represents genes with no difference. (B) GO enrichment analysis of DEGs. (C) KEGG enrichment analysis of DEGs.

**Figure S3. Changes in survival status and survival risk of ccRCC patients with risk score.**

**
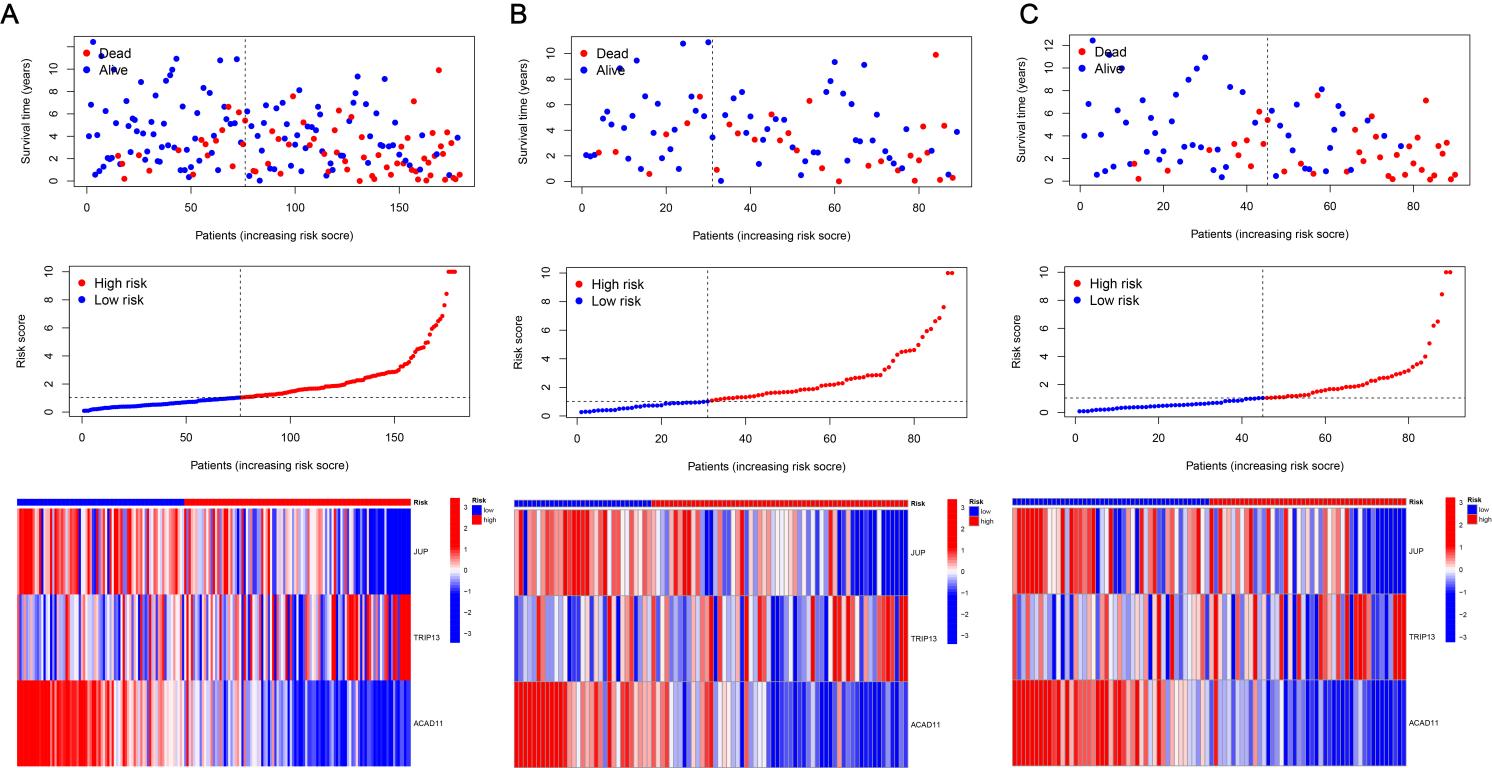
Figure S3. Changes in survival status and survival risk of ccRCC patients with risk score.** (C) All group, (D) Test group, (E) Train group.

**Figure S4. This is a heatmap of immune cell correlation.**


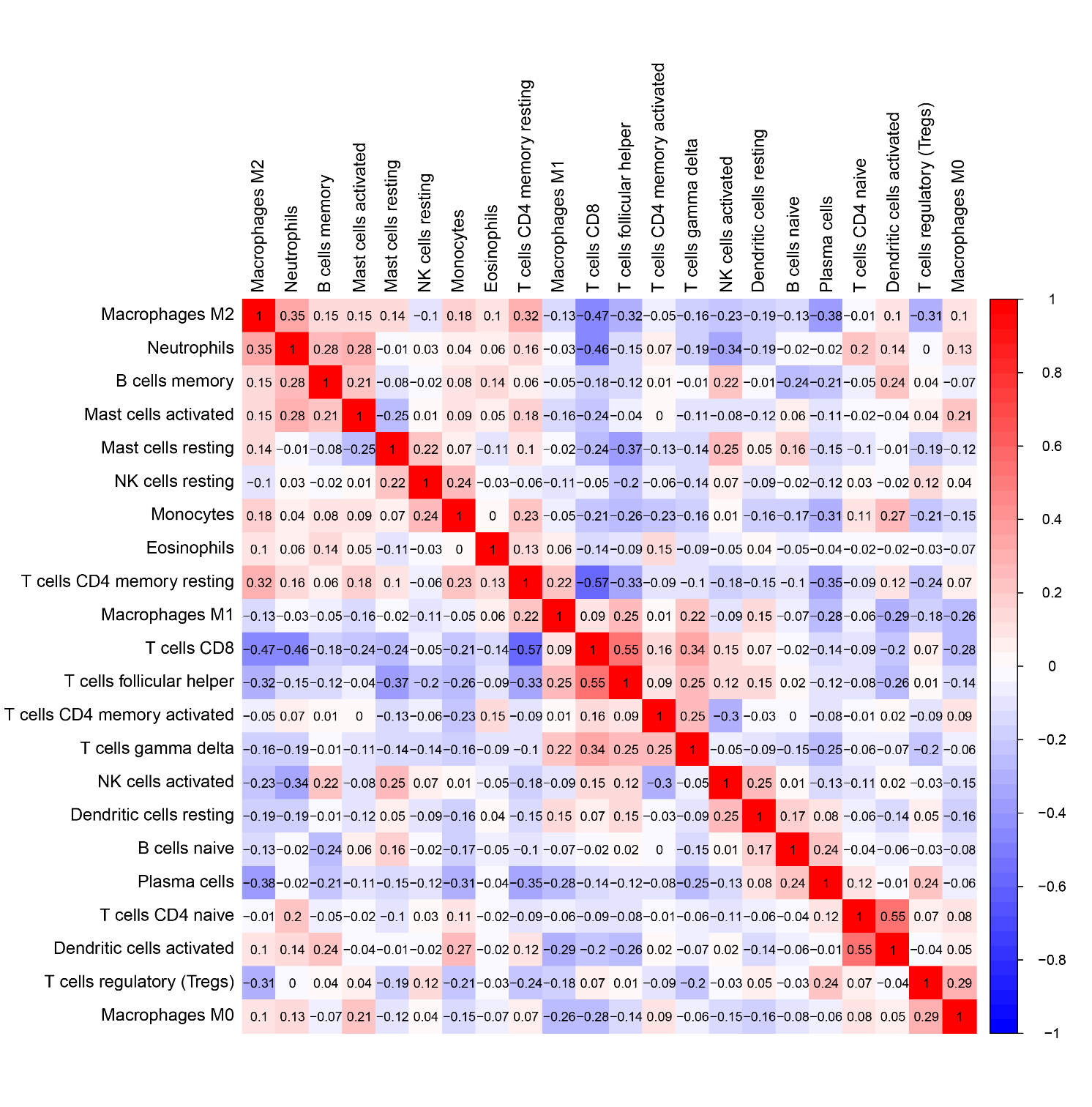


**Figure S4. This is a heatmap of immune cell correlation.** Red represents positive correlation, blue represents negative correlation, the stronger the correlation, the greater the value.

**Figure S5. Correlation between 8 immune cells and risk scores.**


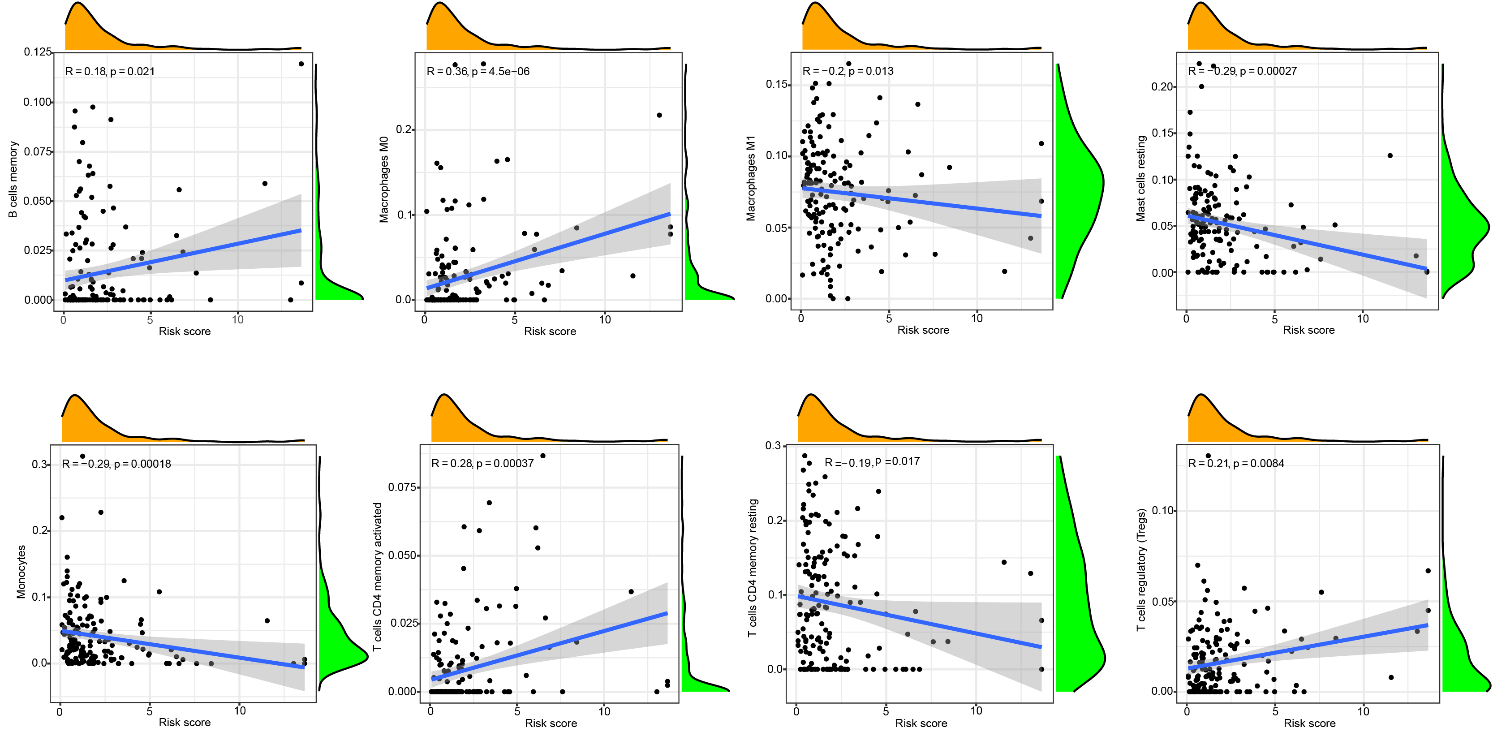


**Figure S5. Correlation between 8 immune cells and risk scores.** R > 0 indicates a positive correlation between immune cells and risk scores, and R < 0 indicates a negative correlation between immune cells and risk scores. *p* < 0.05 indicates a significant difference.

**Figure S6. Biological network integration of TRIP13 analyzed by GeneMANIA.**

**
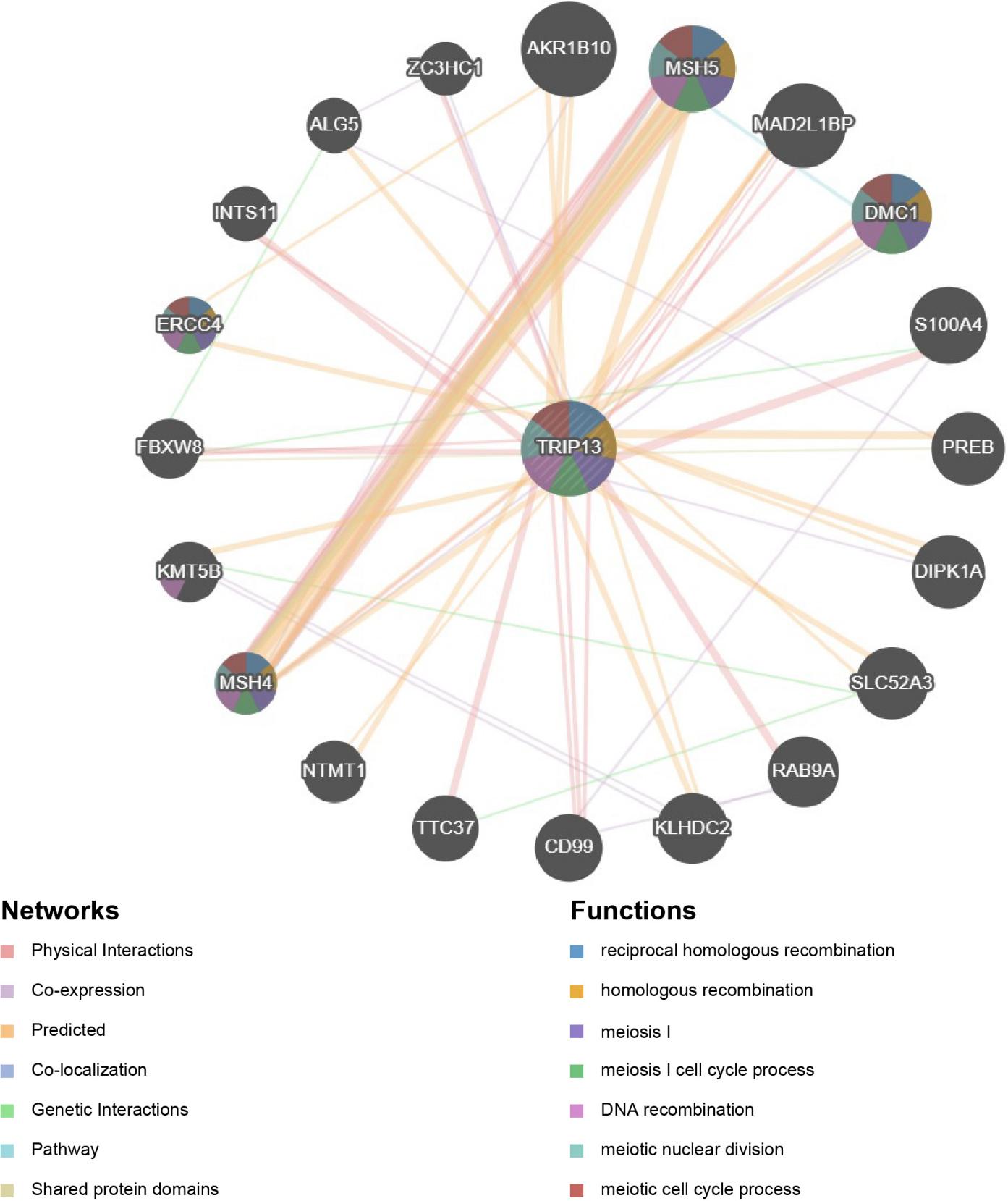
**

**Figure S6. Biological network integration of TRIP13 analyzed by GeneMANIA.** The figure shows the 20 most frequently changing neighboring genes of TRIP13. Each node represents a gene. The color of the node represents the possible function of the corresponding gene, and the lines between genes represent the association between genes.

**Figure S7. hsa-miR-92b-3p is an upstream regulatory mRNA of TRIP13.**


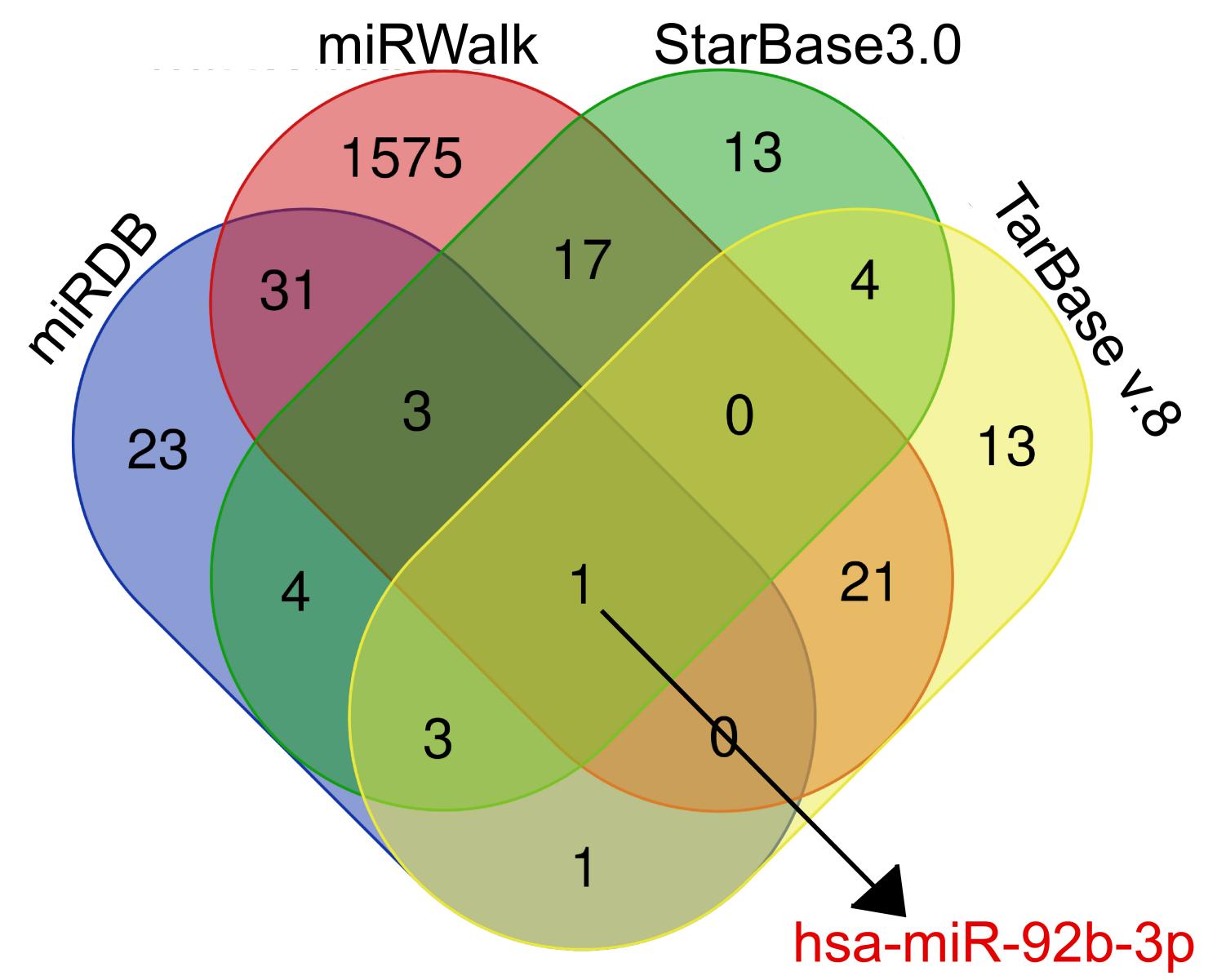


**Figure S7. hsa-miR-92b-3p is an upstream regulatory mRNA of TRIP13.** Four online miRNA prediction databases, miRTarBaseV8.0, StarBase3.0, miRDB and miRWalk, were used to screen the target miRNAs of TRIP13. The Venn diagram showed that there was an overlap between miRNA hsa-miR-92b-3p.

**Supplementary Tables 1-4**

**Table S1.** Learn about the TCGA cohort's ccRCC patients' clinical features.

| Clinical characters | Number |
| --- | --- |
| Gender  Male  Female | 346  191 |
| Age |  |
| Mean (SD) | 60.6 (12.1) |
| Median [MIN, MAX] | 61 [26, 90] |
| TNM stage  I  II  III  IV  X | 269  57  125  83  3 |
| pT_stage  T1  T2  T3  T4 | 275  69  182  11 |
| pN_stage  N0  N1  NX | 240  17  280 |
| pM_stage  M0  M1  MX | 426  79  32 |

**Table S2.** We grabbed 1686 genes from the GeneCards database.

| Gene |
| --- |
| PRKN |
| PINK1 |
| MAP1LC3B |
| VDAC1 |
| FUNDC1 |
| MFN2 |
| SQSTM1 |
| MAP1LC3A |
| ULK1 |
| UBC |
| PHB2 |
| ATG13 |
| SOD2-OT1 |
| TOMM20 |
| AMBRA1 |
| VPS13C |
| PGAM5 |
| MFN1 |
| UBA52 |
| CLEC16A |
| IRGM |
| USP30 |
| DNM1L |
| ATG5 |
| UBB |
| TOMM40 |
| RPS27A |
| OPTN |
| HUWE1 |
| TOMM70 |
| SLC25A4 |
| TOMM7 |
| CSNK2A1 |
| RIMOC1 |
| USP35 |
| SLC25A5 |
| ATG7 |
| ATG4B |
| TOMM22 |
| FBXO7 |
| BNIP3L |
| RNF41 |
| PHB1 |
| TIGAR |
| ABCE1 |
| CSNK2A2 |
| BECN1 |
| BNIP3 |
| VPS13D |
| GABARAPL2 |
| TAFAZZIN |
| ATG4D |
| FIS1 |
| USP15 |
| LRBA |
| RAB7A |
| RHOT1 |
| CERS1 |
| CSNK2B |
| TP53 |
| FKBP8 |
| CHUK |
| ATG12 |
| MIR7-3HG |
| ARFIP2 |
| CALCOCO2 |
| GABARAP |
| ATG4A |
| VDAC2 |
| EMSLR |
| OPA1 |
| ATG9A |
| TBK1 |
| BDNF-AS |
| MAP1LC3B2 |
| SPATA33 |
| CNOT4 |
| MUL1 |
| H19 |
| CISD1 |
| PELO |
| TRIM27 |
| MTX1 |
| TOMM5 |
| SNCA |
| CERNA3 |
| VCP |
| PRKAA1 |
| MTERF3 |
| SRC |
| TOMM6 |
| LRRK2 |
| ATAD3A |
| ATG4C |
| BCL2L13 |
| MTARC2 |
| HDAC6 |
| PTRH2 |
| LINC01554 |
| SIRT1 |
| SAMM50 |
| UBE2L3 |
| MAP2K1 |
| LINC01672 |
| MAP2K2 |
| MON1A |
| WDR26 |
| MDH1 |
| HSPA8 |
| IMMT |
| VDAC3 |
| STK4 |
| BCL2L1 |
| HTRA2 |
| NBR1 |
| OGT |
| LRPPRC |
| NLRP3 |
| MAPK14 |
| ATG14 |
| MFF |
| LINC-PINT |
| TUFM |
| FANCC |
| MAP1LC3C |
| BCAS3 |
| GBA1 |
| PHAF1 |
| TDRKH |
| USP8 |
| HK2 |
| CHCHD3 |
| NOD2 |
| PDK1 |
| TRAP1 |
| ATP1B1 |
| CANX |
| MARCHF5 |
| SMAD5-AS1 |
| PI4KB |
| WIPI1 |
| SESN2 |
| TMX2-CTNND1 |
| MTX2 |
| SPATA18 |
| BAG5 |
| HSP90AA1 |
| SCARNA5 |
| TRA-TGC7-1 |
| PTEN |
| HIF1A |
| UBXN6 |
| GABARAPL1 |
| HSP90AB1 |
| MRPS2 |
| NIPSNAP1 |
| PIP4K2C |
| RNU6-1 |
| ABCD3 |
| CDC37 |
| KRT15 |
| PEX13 |
| MAPK1 |
| ALB |
| PI4K2A |
| STOM |
| STX10 |
| TBC1D5 |
| HSPD1 |
| BCL2 |
| TBC1D15 |
| UQCRC2 |
| EEF1A1 |
| RPL12 |
| MTPAP |
| RPS3 |
| ATP6V1A |
| MIEF1 |
| RPL18 |
| UQCRC1 |
| VPS35 |
| WIPI2 |
| PARL |
| FASN |
| LDHA |
| VWA8 |
| YWHAZ |
| CTPS1 |
| GLS |
| PAICS |
| PLSCR1 |
| RPS5 |
| CYP51A1 |
| GDI2 |
| SNX2 |
| STT3B |
| ATAD3B |
| ALDH2 |
| PGK2 |
| NFE2L2 |
| PGR-AS1 |
| SIRT3 |
| UBE2N |
| MAVS |
| ATG16L1 |
| HSPA9 |
| LMAN1 |
| SDHA |
| TUBB |
| MTOR |
| FOXO3 |
| TSG101 |
| AKT1 |
| AMFR |
| EIF2B1 |
| NIPSNAP2 |
| STOML2 |
| ACIN1 |
| BAG6 |
| CRNKL1 |
| EIF2S1 |
| FXR2 |
| NUP93 |
| PFKP |
| TSPO |
| IDH2 |
| RB1CC1 |
| TARDBP |
| TFRC |
| TSC2 |
| HSPB1 |
| RNF31 |
| CHAF1B |
| MRPL37 |
| PARP1 |
| SUCLA2 |
| TIMM44 |
| UBA1 |
| ATM |
| CLINT1 |
| HK1 |
| MEG3 |
| NDUFV1 |
| RETREG1 |
| SRSF4 |
| UBXN1 |
| VAPA |
| LONP1 |
| MAP2K7 |
| MAPK9 |
| MON2 |
| PRPF8 |
| UBE2D3 |
| ARMCX3 |
| OCIAD1 |
| SIAH3 |
| VIM |
| DSP |
| FADS2 |
| MIF |
| SLC25A24 |
| STEAP3 |
| TOM1 |
| ANXA5 |
| EMC1 |
| FBXL4 |
| FBXW7 |
| HNRNPM |
| HSPA1A |
| MAP2K3 |
| NAA10 |
| NAA16 |
| NAXE |
| PRKAA2 |
| PRKCD |
| SCAMP3 |
| SEC22B |
| ACTB |
| APP |
| HNRNPH1 |
| HNRNPU |
| PDIA6 |
| PKM |
| TUBA1C |
| TUBB4B |
| TUBB6 |
| BLOC1S1 |
| CCZ1B |
| GPD2 |
| HILPDA |
| HSPA1L |
| RMDN3 |
| ARIH1 |
| CCT3 |
| FUS |
| GAPDH |
| HSP90B1 |
| IMPDH2 |
| MYH9 |
| NPLOC4 |
| PFAS |
| RAB1B |
| RPL7 |
| ABCB10 |
| CCT4 |
| CFL1 |
| EPRS1 |
| HNRNPD |
| MCM7 |
| NUP155 |
| PLEC |
| RPL11 |
| RPL21 |
| RPL8 |
| RPLP0 |
| RPS15A |
| RPS18 |
| RPS2 |
| RPS3A |
| SHMT2 |
| SPTBN1 |
| TIMM23 |
| TRIM25 |
| YWHAE |
| ACTR1A |
| CAND1 |
| CCT7 |
| CSE1L |
| DAP3 |
| DARS2 |
| EEF1G |
| EEF2 |
| EIF2AK2 |
| EIF3F |
| EIF4A1 |
| EIF4A3 |
| ESYT1 |
| GANAB |
| GLUD1 |
| GMPPA |
| IDH3A |
| MTCH2 |
| NCAPD2 |
| PRKDC |
| PYCR1 |
| RAN |
| RFC3 |
| ROCK2 |
| RPL4 |
| RPL9 |
| RPS9 |
| RUVBL1 |
| SARS1 |
| SF3A1 |
| TRAF6 |
| TUBB4A |
| UMPS |
| YWHAH |
| NFKB1 |
| AAAS |
| ACTBL2 |
| BCKDK |
| C9orf72 |
| CCDC47 |
| CNP |
| CORO1C |
| CTNND1 |
| DDX39B |
| EIF4A2 |
| ENO1 |
| ESYT2 |
| FAF2 |
| HS2ST1 |
| JUP |
| MAPK8 |
| NPEPPS |
| PHGDH |
| PIGK |
| PREB |
| RPL15 |
| RPL28 |
| SEC23A |
| SMARCAD1 |
| TMPO |
| TUBG1 |
| WDR41 |
| XPO1 |
| ZC3HAV1 |
| ACLY |
| ACSF3 |
| ACSL4 |
| ACTR2 |
| ADSS2 |
| AHCY |
| ALDH18A1 |
| ALDOA |
| ATP5F1B |
| CAT |
| CDK11A |
| CHKB-CPT1B |
| COASY |
| EPB41L3 |
| ERLIN2 |
| FDPS |
| FEN1 |
| GDI1 |
| GFPT2 |
| IDH3B |
| ILF2 |
| ILK |
| LAP3 |
| MAP3K7 |
| MRI1 |
| MRPS30 |
| NAMPT |
| OAT |
| PCNA |
| PGM1 |
| PRKCI |
| RPL18A |
| SEC24A |
| SERPINB6 |
| SF3A2 |
| SRP54 |
| TMEM43 |
| TRIP13 |
| TUBB2B |
| UCHL1 |
| UGDH |
| UTP18 |
| WARS1 |
| ARIH2 |
| BLMH |
| MCL1 |
| MIR320A |
| ACBD3 |
| ALDH3A2 |
| ATP6AP1 |
| CDK5RAP3 |
| CKB |
| CORO1A |
| DCTN4 |
| FBXW11 |
| FIG4 |
| GBF1 |
| GDAP1 |
| GNAS |
| GOT2 |
| GPHN |
| GSK3A |
| KDELR1 |
| LBR |
| NR4A1 |
| OSBPL11 |
| OSBPL9 |
| PDHA1 |
| PGK1 |
| PIGT |
| PPP3CA |
| PTPN1 |
| RMST |
| RRAGC |
| SEH1L |
| SGPL1 |
| SLC33A1 |
| SNX1 |
| SNX30 |
| SRPRA |
| STIM1 |
| TBC1D22A |
| U2AF2 |
| UVRAG |
| VPS45 |
| ATP6V0D1 |
| RRM1 |
| BET1 |
| HTT |
| NDUFA10 |
| ZFYVE16 |
| FAM20B |
| ATG2B |
| HDHD5 |
| MAPK15 |
| TFEB |
| TMEM11 |
| TRC-GCA24-1 |
| CHDH |
| MST1 |
| SLC25A13 |
| AGK |
| SEC61A1 |
| MT-TK |
| TGFB1 |
| ACO2 |
| AIFM1 |
| PIK3C3 |
| AARS1 |
| ACAD11 |
| AKR1E2 |
| ANXA1 |
| APEH |
| ATP6V1G1 |
| CPVL |
| FH |
| LMCD1 |
| MAP1A |
| MIR138-2 |
| MYH11 |
| MYLK |
| PMPCB |
| PRKD2 |
| RHOT2 |
| RIPK1 |
| SMURF1 |
| TFE3 |
| VTRNA1-1 |
| YARS1 |
| C1QBP |
| NME4 |
| PSMA7 |
| PSMB5 |
| STARD7 |
| TCHP |
| TXN |
| USP36 |
| ALDH7A1 |
| CAV1 |
| CKAP4 |
| HNRNPA2B1 |
| PMPCA |
| PYCR1-AS1 |
| RAD23B |
| RMC1 |
| SHC1 |
| SNHG14 |
| STX17 |
| ZBTB17 |
| CDKN2A |
| IDH3G |
| NME1 |
| RNF121 |
| UBE2G2 |
| USP33 |
| ACTRT1 |
| ANXA2 |
| BIRC2 |
| CLPB |
| DCD |
| DSG2 |
| IRF3 |
| KIAA0232 |
| LMO7 |
| MTARC1 |
| NADSYN1 |
| PLOD2 |
| RFWD3 |
| SPTBN2 |
| TRAF2 |
| TUG1 |
| VAPB |
| VPS29 |
| XIST |
| ACAA2 |
| CREB1 |
| DHFR |
| DNAJC3 |
| DPYSL5 |
| EARS2 |
| EDC3 |
| EDC4 |
| HNRNPDL |
| HSPA4 |
| L2HGDH |
| MAPK3 |
| MON1B |
| MRPS34 |
| NDUFAF4 |
| POLR2H |
| PPIB |
| PPM1G |
| PRDX1 |
| PSMB2 |
| PTPMT1 |
| RAB10 |
| UBAP2L |
| USP14 |
| AKAP1 |
| ANO6 |
| ARL6IP5 |
| CHMP2A |
| EPHA2 |
| EPS15 |
| ITCH |
| KHNYN |
| LPCAT3 |
| MIR155 |
| MIR302A |
| MT-ND6 |
| SAR1A |
| SLC12A4 |
| SLC12A6 |
| TBC1D17 |
| ADH5 |
| APBB1 |
| AUP1 |
| B3GAT3 |
| CAPN1 |
| CDC34 |
| CHMP5 |
| CYP20A1 |
| DISC1 |
| FADS3 |
| FAF1 |
| FBL |
| GLT8D1 |
| HLA-C |
| HPF1 |
| KEAP1 |
| LYAR |
| PNO1 |
| PPARGC1A |
| RPS26 |
| RPTOR |
| SEC61B |
| SEC62 |
| SENP3 |
| SNAP47 |
| SPTAN1 |
| SRPRB |
| TAX1BP1 |
| TCP1 |
| VCAM1 |
| VPS26B |
| ATG2A |
| EMC4 |
| REEP5 |
| SAR1B |
| SH3GLB1 |
| SNX3 |
| SPG21 |
| YKT6 |
| ABAT |
| ANKRD13A |
| ARL8B |
| ARMCX2 |
| ARPC4 |
| BLVRA |
| CD55 |
| CFAP20 |
| DCXR |
| DERA |
| DHX29 |
| DPM1 |
| EMD |
| LRRC59 |
| MACROH2A1 |
| MAGOHB |
| MRPL4 |
| MRPL44 |
| MRPS16 |
| NDUFA9 |
| NDUFS7 |
| NME6 |
| NUP205 |
| PGAM1 |
| PIP4K2B |
| PLEKHA1 |
| PSMG3 |
| RAP1B |
| RPL13A |
| RPL35A |
| RPL38 |
| RPS27L |
| RTN4 |
| SHPK |
| TNFAIP8L1 |
| TRAPPC12 |
| VPS37A |
| WDR6 |
| AARS2 |
| ACTR10 |
| AKAP11 |
| ATP5IF1 |
| ATP7B |
| BCAT2 |
| BLTP3A |
| CALU |
| CISD2 |
| CLUH |
| COG6 |
| DEF8 |
| DHX38 |
| DIAPH2 |
| EXD2 |
| FKBP15 |
| FLOT2 |
| FNIP2 |
| FYCO1 |
| GCC1 |
| GFAP |
| GLUL |
| GOLGA4 |
| GOLGA5 |
| GOLGB1 |
| GOPC |
| HS1BP3 |
| ITPR2 |
| KPNA5 |
| LRCH4 |
| MADD |
| MBOAT7 |
| MEGF8 |
| MT-RNR1 |
| NDC1 |
| NDUFS1 |
| PCK2 |
| PIK3R4 |
| PPFIBP1 |
| PRKACA |
| PRKD3 |
| RABGAP1 |
| RALGAPA1 |
| RRAGA |
| RTN3 |
| SCRN1 |
| SEC16A |
| SEC24B |
| SEC63 |
| SIRT2 |
| SLC12A2 |
| SMAD2 |
| SNX17 |
| SNX4 |
| SOAT1 |
| SREBF1 |
| SREBF2 |
| ST7 |
| TOR1AIP1 |
| TRIP11 |
| TUBA8 |
| UBE2Z |
| VAMP7 |
| VIPAS39 |
| VPS26A |
| VPS26C |
| VPS41 |
| WASHC4 |
| WWOX |
| ZDHHC13 |
| APAF1 |
| ARF4 |
| ARPC2 |
| CPT1A |
| CYB5R1 |
| DDX54 |
| EIF3C |
| HMGB1 |
| NFKB2 |
| PSMD8 |
| PVT1 |
| SLC3A2 |
| SPCS2 |
| TEX10 |
| TMX3 |
| TNF |
| TOR1AIP2 |
| CCT2 |
| DBN1 |
| FTMT |
| MYO6 |
| NKAP |
| BCS1L |
| CA2 |
| CWC22 |
| EWSR1 |
| FASTKD5 |
| GRN |
| HDGF |
| IGF2R |
| NEPRO |
| NIBAN2 |
| PAK2 |
| PITRM1 |
| PUM2 |
| RCN1 |
| SAE1 |
| TAB1 |
| TALDO1 |
| THOP1 |
| VPS16 |
| ACTL6A |
| CDKAL1 |
| DGKE |
| DHX57 |
| GORASP2 |
| KIF1A |
| MIOS |
| MT-TS1 |
| NEFM |
| NRF1 |
| OSBPL8 |
| PIGS |
| POLR3A |
| POR |
| RCAN1 |
| SCD |
| SDCBP |
| SIAH1 |
| SUN1 |
| SYNE2 |
| TMEM256-PLSCR3 |
| U2AF1 |
| UBE2D2 |
| VAC14 |
| YME1L1 |
| MYO1B |
| PRDX6 |
| TRNT1 |
| ZFYVE1 |
| HDAC3 |
| ATP4A |
| RAB11FIP5 |
| RAB4B-EGLN2 |
| SCFD1 |
| STX12 |
| KRAS |
| WDR46 |
| ATAD3C |
| ATP5F1A |
| FMR1 |
| HRAS |
| IBA57 |
| LANCL1 |
| PIN1 |
| PNP |
| BAX |
| GLB1 |
| IGF1 |
| LMNA |
| MIR379 |
| SLC25A12 |
| SLC25A3 |
| UBE2D1 |
| XRCC6 |
| DDX17 |
| DDX5 |
| HNRNPA3 |
| HNRNPK |
| HSPA5 |
| MATR3 |
| PARK7 |
| RPS16 |
| RPS6 |
| MIR106B |
| MIR25 |
| MIR93 |
| PDK2 |
| XIAP |
| ACACA |
| ACSL3 |
| AFG3L2 |
| ARCN1 |
| CALR |
| DDX3X |
| DHX9 |
| DIS3 |
| DNAJA3 |
| DNAJC7 |
| ERAL1 |
| GAK |
| HNRNPA1 |
| HNRNPC |
| HNRNPF |
| HNRNPR |
| IARS2 |
| IGF2BP1 |
| IGKC |
| IRAK1 |
| LIG3 |
| LMNB1 |
| MCCC2 |
| MCM4 |
| MCM6 |
| MCU |
| MIR27A |
| MRPS7 |
| NDUFS2 |
| OSBPL5 |
| PABPC1 |
| PSMC1 |
| PSMD1 |
| PSMD3 |
| PSMD4 |
| PTCD3 |
| RANBP2 |
| RDH13 |
| RPL13 |
| RPL22 |
| RPL5 |
| RPN1 |
| RPS14 |
| RPS8 |
| SFPQ |
| SNRNP200 |
| STK38 |
| SYNCRIP |
| UFL1 |
| WRNIP1 |
| ABCF2 |
| ACTN4 |
| BAG2 |
| BAG3 |
| CAD |
| CCT6A |
| CLTC |
| DNAJA1 |
| DYNC1H1 |
| HDLBP |
| HNRNPH2 |
| HSD17B10 |
| IGF2BP3 |
| MCM2 |
| MCM5 |
| MTHFD1 |
| NCL |
| PPP2R1A |
| PSMC6 |
| PSMD11 |
| PSMD13 |
| PSMD2 |
| PSMD6 |
| RACK1 |
| RBBP4 |
| RBM14 |
| RBM39 |
| RPL10 |
| RPL14 |
| RPL17 |
| RPL6 |
| RPL7A |
| RPS20 |
| RPS25 |
| RPSA |
| RUVBL2 |
| SLC25A1 |
| WDR18 |
| WDR77 |
| BAK1 |
| CHCHD2P9 |
| HCCS |
| HSPA6 |
| IKBKG |
| LINC02208 |
| LINC02517 |
| LINC-ROR |
| LOC105378097 |
| MT-RNR2 |
| MT-TL1 |
| SCO2 |
| UBE2D4 |
| UBE2E3 |
| ACADSB |
| ACTR3 |
| ADPGK |
| AFG2A |
| AGPS |
| AHSA1 |
| ALG1 |
| ASNS |
| ATIC |
| ATP2A2 |
| ATP6V1H |
| CAMK2D |
| CCT5 |
| CCT8 |
| CDC73 |
| CHD4 |
| CIAO1 |
| COPB1 |
| COPB2 |
| COPZ1 |
| CTTN |
| CUL4A |
| CUL4B |
| DCAF8 |
| DDX19A |
| DDX21 |
| DDX23 |
| DDX47 |
| DDX6 |
| DHRS7B |
| DHX15 |
| DNM2 |
| DPP9 |
| DYNC1I2 |
| EFTUD2 |
| EIF3A |
| EIF3B |
| EIF3L |
| EIF5B |
| ELAVL1 |
| ELP1 |
| EPPK1 |
| ETFB |
| FARSB |
| FKBP4 |
| FLNB |
| FLNC |
| FUBP3 |
| FXR1 |
| GALK1 |
| GARS1 |
| GART |
| GCDH |
| GMPS |
| GNB2 |
| GPI |
| GSPT1 |
| GTF3C4 |
| HADHA |
| HADHB |
| HAT1 |
| HLA-A |
| HNRNPL |
| HSDL2 |
| HSPA4L |
| HYOU1 |
| IARS1 |
| IGF2BP2 |
| ILF3 |
| ILVBL |
| IQGAP1 |
| KARS1 |
| KHDRBS1 |
| KPNA6 |
| LARS1 |
| LDHB |
| LMNB2 |
| MAGED2 |
| MCM3 |
| MCMBP |
| MOV10 |
| MRE11 |
| MRPS27 |
| MTA2 |
| NCLN |
| NONO |
| NOP2 |
| NSF |
| NUP214 |
| NUP88 |
| ORC5 |
| P4HB |
| PA2G4 |
| PABPC4 |
| PANK4 |
| PC |
| PDCD6IP |
| PGS1 |
| POLD1 |
| POLD2 |
| POLR2B |
| PPM1F |
| PPP2R2A |
| PPP2R2D |
| PRMT5 |
| PRPF19 |
| PRPF4B |
| PRPF6 |
| PRPSAP1 |
| PSAT1 |
| PSMC5 |
| RAB21 |
| RARS1 |
| RBBP7 |
| RBM25 |
| RBMX |
| RIC8A |
| RNPEP |
| RO60 |
| RPL19 |
| RPL24 |
| RPL27A |
| RPL29 |
| RPN2 |
| SACM1L |
| SARS2 |
| SEPTIN9 |
| SF3B1 |
| SF3B2 |
| SFXN1 |
| SLC25A22 |
| SLC25A25 |
| SMARCA5 |
| SMC1A |
| SND1 |
| SPTLC1 |
| SPTLC2 |
| SRP68 |
| SRP72 |
| SRRM2 |
| SRSF1 |
| SSB |
| ST13 |
| STIP1 |
| STK26 |
| STRAP |
| STRN4 |
| TLN1 |
| TNPO1 |
| TRAFD1 |
| TRIM28 |
| U2SURP |
| UFSP2 |
| VARS1 |
| VTN |
| YBX1 |
| YWHAG |
| ZC3H15 |
| ZNF326 |
| ABCF3 |
| ACTC1 |
| BBC3 |
| BRIX1 |
| CDC20 |
| CTNNBL1 |
| CYC1 |
| DNAJA2 |
| EIF4G1 |
| FARSA |
| FLT3 |
| GNL2 |
| GNL3 |
| HGH1 |
| HGS |
| HNRNPAB |
| KPNB1 |
| LGALS1 |
| LGALS3BP |
| MIR27B |
| MRPL3 |
| MTFP1 |
| MYBBP1A |
| NAT10 |
| NOP56 |
| NOP58 |
| PAFAH1B1 |
| PCBP1 |
| PCBP2 |
| PDE12 |
| PRCC |
| PRKACB |
| PRMT1 |
| PRPF31 |
| PSMC3 |
| PSMC4 |
| PSMD12 |
| PSMD14 |
| PUF60 |
| RAB35 |
| RFC1 |
| RPL10A |
| RPS11 |
| RPS12 |
| RPS6KA3 |
| SAMHD1 |
| SHMT1 |
| SMARCD1 |
| STUB1 |
| TECR |
| TUBA1B |
| TUBB2A |
| XRN1 |
| BAG4 |
| BST2 |
| GOSR1 |
| MT-ND5 |
| RIGI |
| WDR45 |
| WFS1 |
| AAMP |
| ABCB7 |
| ABCF1 |
| ABHD12 |
| ACAD9 |
| ACADVL |
| ACAT1 |
| ACOT9 |
| ACOX1 |
| ACTN1 |
| ADD3 |
| ADSL |
| AGO2 |
| AHCYL1 |
| AHSG |
| AIMP2 |
| AKAP8L |
| ALDH16A1 |
| ALDH1B1 |
| ALDH5A1 |
| ALDH9A1 |
| ANAPC7 |
| ANKFY1 |
| ANLN |
| AP1B1 |
| AP1G1 |
| AP1M1 |
| AP2A1 |
| AP2M1 |
| AP3B1 |
| AP3D1 |
| AP3M1 |
| APMAP |
| APOB |
| APRT |
| ARHGAP1 |
| ARHGEF1 |
| ARHGEF2 |
| ARMT1 |
| ASMTL |
| ASS1 |
| ATP5PB |
| ATP6V1B2 |
| ATXN10 |
| BABAM2 |
| BCCIP |
| BCKDHA |
| BEX2 |
| BSG |
| BUB3 |
| BYSL |
| BZW1 |
| BZW2 |
| CAP1 |
| CAPN11 |
| CAPN2 |
| CAPZB |
| CARM1 |
| CARS1 |
| CARS2 |
| CBL |
| CBR1 |
| CCAR1 |
| CDC27 |
| CDC5L |
| CDK1 |
| CDK8 |
| CHEK2 |
| CLIC1 |
| CLP1 |
| CLPTM1L |
| CMAS |
| CNDP2 |
| CNN3 |
| COPA |
| COPG1 |
| COQ6 |
| CORO1B |
| CORO7 |
| CPNE1 |
| CPNE3 |
| CPSF2 |
| CPSF6 |
| CRLF3 |
| CRTAP |
| CS |
| CSDE1 |
| CSK |
| CSTF1 |
| CSTF3 |
| CTBP1 |
| CTBP2 |
| CUL2 |
| CUL3 |
| CUL5 |
| DARS1 |
| DAZAP1 |
| DBR1 |
| DDB1 |
| DDB2 |
| DDI2 |
| DDOST |
| DDX1 |
| DDX18 |
| DDX27 |
| DDX39A |
| DDX46 |
| DDX49 |
| DDX52 |
| DDX56 |
| DEK |
| DHX30 |
| DKC1 |
| DLD |
| DLST |
| DNMT1 |
| DNPEP |
| DOCK7 |
| DPP3 |
| DPYSL2 |
| DPYSL3 |
| DRG1 |
| DRG2 |
| DTL |
| DTYMK |
| DYNC1LI1 |
| EED |
| EHD1 |
| EHD4 |
| EIF2B3 |
| EIF2B5 |
| EIF2S2 |
| EIF2S3 |
| EIF3D |
| EIF3E |
| EIF3G |
| EIF3I |
| EIF4G2 |
| EIPR1 |
| ELAC2 |
| ELP3 |
| EML2 |
| EP300 |
| EPB41L5 |
| EPHX1 |
| EPS8L2 |
| ERO1A |
| ETF1 |
| EXOC4 |
| EXOSC10 |
| F13A1 |
| FAM120A |
| FAM98A |
| FAM98B |
| FAR1 |
| FBXL18 |
| FBXO22 |
| FBXO3 |
| FDXR |
| FECH |
| FERMT2 |
| FLII |
| FLOT1 |
| FNTB |
| FSCN1 |
| FTSJ3 |
| G3BP2 |
| G6PD |
| GAPVD1 |
| GATAD2B |
| GCLC |
| GFM1 |
| GFPT1 |
| GHDC |
| GMDS |
| GNA11 |
| GNA13 |
| GNAI2 |
| GNL1 |
| GOT1 |
| GPCPD1 |
| GPS1 |
| GPSM1 |
| GRHPR |
| GRWD1 |
| GSTP1 |
| GTF3C1 |
| GTF3C5 |
| GTPBP1 |
| GYS1 |
| HACD3 |
| HARS2 |
| HAUS4 |
| HBD |
| HBS1L |
| HDAC1 |
| HDAC2 |
| HMBS |
| HMGCS1 |
| HNRNPUL1 |
| HP1BP3 |
| HPRT1 |
| HSD17B12 |
| HSPA13 |
| HSPH1 |
| IDH1 |
| IGHG2 |
| IPO5 |
| IVD |
| JMJD6 |
| KDM3B |
| KIF11 |
| KIF3A |
| KIF3B |
| KIF5B |
| KMT2C |
| KPNA2 |
| KPNA4 |
| KYAT3 |
| LANCL2 |
| LARS2 |
| LDB1 |
| LLGL1 |
| LONP2 |
| LRCH2 |
| LRRC40 |
| LRRC47 |
| LTA4H |
| LUC7L2 |
| LUC7L3 |
| MAEA |
| MAGT1 |
| MAP4K4 |
| MAPKAPK2 |
| MARS1 |
| MAT2A |
| MBNL2 |
| METAP1 |
| METTL17 |
| MIR193B |
| MIR210 |
| MKRN2 |
| MMS19 |
| MRPS5 |
| MRPS9 |
| MSRB2 |
| MT-CO2 |
| MTMR9 |
| MYO9B |
| NAA15 |
| NADK2 |
| NAE1 |
| NAP1L1 |
| NARF |
| NARS1 |
| NARS2 |
| NCKAP1 |
| NDRG1 |
| NEDD4L |
| NELFB |
| NFS1 |
| NKRF |
| NLE1 |
| NMD3 |
| NR1D1 |
| NSDHL |
| NSUN2 |
| NT5C2 |
| NT5DC2 |
| NUDCD1 |
| NUDT19 |
| NUP54 |
| NUP85 |
| NUP98 |
| NXN |
| OAS3 |
| OCRL |
| OLA1 |
| OSBP |
| OXCT1 |
| OXSR1 |
| P4HA1 |
| P4HA2 |
| PAF1 |
| PALLD |
| PAPSS1 |
| PAPSS2 |
| PARD6B |
| PATL1 |
| PCYOX1 |
| PDIA3 |
| PDK3 |
| PDLIM5 |
| PDP1 |
| PES1 |
| PFKL |
| PFKM |
| PGD |
| PGM2 |
| PGM3 |
| PHF6 |
| PICALM |
| PKN1 |
| PKN2 |
| PLAA |
| PLCG1 |
| PLRG1 |
| PM20D2 |
| PMS2 |
| POFUT1 |
| POLA2 |
| POLR1C |
| POLR3B |
| PPAT |
| PPFIA1 |
| PPIL4 |
| PPME1 |
| PPOX |
| PPP1R7 |
| PPP2R5C |
| PPP5C |
| PPWD1 |
| PRDX4 |
| PREP |
| PRIM1 |
| PRIM2 |
| PRKAG1 |
| PRKAR1A |
| PRKAR2A |
| PRMT3 |
| PRPF3 |
| PRPF38B |
| PRPF4 |
| PRPS1 |
| PSPC1 |
| PTBP1 |
| PTGES3 |
| PTPN11 |
| PUS1 |
| PWP1 |
| PYGB |
| PYGL |
| QARS1 |
| RACGAP1 |
| RAD9A |
| RANGAP1 |
| RBBP5 |
| RBM12 |
| RBM22 |
| RCC1L |
| RCC2 |
| RECQL |
| RELA |
| RFC2 |
| RFC5 |
| RHOA |
| RNH1 |
| RPA1 |
| RPL3 |
| RPL32 |
| RPL34 |
| RPL36 |
| RPS17 |
| RPS23 |
| RPS4X |
| RPS6KA1 |
| RSL1D1 |
| RTCB |
| RTF1 |
| SCRIB |
| SCYL1 |
| SEC23B |
| SEC24C |
| SEC31A |
| SELENBP1 |
| SEPTIN10 |
| SEPTIN11 |
| SEPTIN2 |
| SEPTIN7 |
| SEPTIN8 |
| SERPINB1 |
| SERPINH1 |
| SF1 |
| SKP2 |
| SLC16A1 |
| SLC25A10 |
| SLC2A3 |
| SLFN11 |
| SMAD3 |
| SMARCD2 |
| SMS |
| SMU1 |
| SNRNP40 |
| SNRNP70 |
| SNTB2 |
| SNX27 |
| SNX6 |
| SNX9 |
| SORD |
| SRPK1 |
| SRRT |
| SRSF6 |
| SSRP1 |
| STAM2 |
| STAT3 |
| STT3A |
| STXBP1 |
| SUDS3 |
| SWAP70 |
| TAF5 |
| TARS1 |
| TARS2 |
| TBC1D24 |
| TBC1D4 |
| TBCE |
| TBL1XR1 |
| TBL2 |
| TBRG4 |
| TES |
| TFG |
| THOC2 |
| THUMPD1 |
| THUMPD3 |
| TJP2 |
| TLK2 |
| TM9SF3 |
| TMEM33 |
| TOP1 |
| TPP1 |
| TPP2 |
| TPT1 |
| TRA2B |
| TRIM21 |
| TRIM26 |
| TRIP6 |
| TRMT1 |
| TRMT10C |
| TRMT5 |
| TRMU |
| TSEN2 |
| TSR1 |
| TTC38 |
| TTLL12 |
| TUBA1A |
| TUBAL3 |
| TUBGCP3 |
| UAP1L1 |
| UBA2 |
| UBA3 |
| UBLCP1 |
| UBR4 |
| UCKL1 |
| UGP2 |
| UPF1 |
| UPF2 |
| USP39 |
| USP47 |
| USP9X |
| WDFY1 |
| WDHD1 |
| WDR1 |
| WDR12 |
| WDR70 |
| XRCC5 |
| XRN2 |
| YARS2 |
| YES1 |
| YTHDC1 |
| YTHDC2 |
| YWHAB |
| ZC3H18 |
| ZNF277 |
| ZNF512 |
| ZNF622 |
| A1BG |
| A2M |
| ACTG1 |
| ADAM17 |
| CD81 |
| CDIPT |
| CLPP |
| COX15 |
| GADD45GIP1 |
| IL24 |
| IL25 |
| KIAA1671 |
| LAMP2 |
| LINC00520 |
| MIR181A1 |
| MIRLET7B |
| MIRLET7C |
| MLF2 |
| MUC1 |
| NBN |
| NEXN |
| SPECC1 |
| SPHK2 |
| TMEM165 |
| TRIM5 |
| TRN-GTT2-1 |
| VPS13A |
| DUSP1 |
| IAPP |
| TGM2 |
| UBE2A |
| UBE2J2 |

**Table S3.** Multivariate Cox results selected three MRGs.

| MRGs | Coef |
| --- | --- |
| JUP | -0.439181231277887 |
| TRIP13 | 0.471404750400818 |
| ACAD11 | -1.21854543241319 |

MRGs, mitophagy-related genes; Coef, coefficient.

**Table S4.** Detailed IC50-values for the 60 drugs.
